# Regulation of DNA Methylation in Peanut Leaves and Roots: Uncovering the Molecular Mechanisms for Increased Yield After Single‐Seed Sowing

**DOI:** 10.1111/pbi.70264

**Published:** 2025-07-22

**Authors:** Shikai Fan, Jialei Zhang, Feng Guo, Fangji Xu, Zhaohui Tang, Rongchong Li, Bo Bai, Yiyang Liu, Guowei Li, Shubo Wan

**Affiliations:** ^1^ Institute of Crop Germplasm Resources Shandong Academy of Agricultural Sciences Ji'nan China

**Keywords:** DNA methylation, flavonoid, leaf senescence, metagenomics, nodule, peanut, single‐seed precision sowing

## Abstract

Cytosine methylation is a crucial epigenetic modification that responds to various environmental cues, yet the specific mechanisms influencing planting patterns remain incompletely understood. This study reveals significant growth differences between single‐seed (SS) precision sowing and double‐seed (DS) sowing observed 42 days after germination under controlled indoor conditions. These differences were eliminated by the application of the DNA methylation inhibitor 5‐azacytidine (5‐aza), highlighting the role of DNA methylation in these processes. To further investigate the role of DNA methylation in planting pattern, we generated DNA methylation profiles of peanut leaves and roots under both DS and SS planting patterns. The analysis revealed increased CHH methylation in both tissues, caused by the RNA‐directed DNA methylation (RdDM) pathway. Further analysis, including differential methylation, transposable element (TE) analysis and methylation‐related gene analysis, demonstrated tissue‐specific epigenetic responses to planting patterns. Integrating methylome and transcriptome data, we found that DS was associated with hyper‐CHH methylation in WRKY gene promoters in leaves, accelerating leaf senescence. Meanwhile, SS reduced CHH methylation in promoters in roots, upregulating genes involved in flavonoid biosynthesis. This upregulation enhanced root nodule formation and improved stress resistance, resulting in increased concentrations of nitrogen and phosphorus in the roots, as confirmed by metagenomic functional analysis. This research provides novel insights into the epigenetic regulation of plant growth and development.

## 1 Introduction

Peanuts are a major oil, economic and distinctive export crop in China. With the increasing scarcity of arable land and the growing competition between grain and oil crops for land use, it has become clear that increasing yield is a crucial and effective strategy to boost peanut production. Although the traditional method of double‐seed (DS) sowing (i.e., sowing two seeds per hole) ensures a high germination rate, it also intensifies competition among plants, limiting individual growth potential and hindering yield improvement. To address this issue, our team proposed the single‐seed (SS) precision sowing for optimising population yield (Zhang et al. 2020). This technology reduces interplant competition, saves 20% of seeds, achieves a yield increase of over 10% and promotes the healthy development of the peanut industry by increasing farmers' income (Zhang et al. 2020).

To elucidate the mechanisms behind the yield increase and efficiency enhancement provided by this technology, our research team has conducted extensive physiological and molecular‐level studies (Yang et al. 2023, 2021; Zhang et al. 2020). Physiologically, SS sowing enhances leaf photosynthetic efficiency, promotes the accumulation of photosynthetic products and nutrient uptake, delays leaf senescence, improves the microenvironment of the peanut canopy, promotes root morphological development and enhances the overall quality of the peanut population during the growth period (Liang et al. 2020; Zhang et al. 2020). Previous transcriptomic analyses have shown that SS precision sowing enhances peanut yield compared to DS sowing by improving root resistance to *Aspergillus flavus* and abiotic stresses. Furthermore, in contrast to conventional multiseed sowing (three or more seeds per hole), SS sowing has been reported to increase leaf photosynthetic efficiency and strengthen root responses to both biotic and abiotic challenges (Yang et al. 2023, 2021). While transcriptome analyses have identified numerous differentially expressed genes associated with these planting strategies, the underlying transcriptional and epigenetic regulatory mechanisms remain incompletely understood. Given that the genetic material is identical between individuals in the two planting patterns, the observed growth differences are likely due to variations in gene expression, implicating epigenetic regulation as a key factor. Epigenetic mechanisms, such as DNA methylation (5‐methylcytosine, 5mC), play a crucial role in regulating gene expression and cellular functions without altering the underlying DNA sequence. DNA methylation, a highly conserved modification across evolution, occurs in plants within three specific sequence contexts: CG, CHG and CHH (where H represents A, C, or T) (Gallego‐Bartolome 2020; Law and Jacobsen 2010; Zhang et al. 2018). The homeostasis of DNA methylation in plants is tightly controlled through three main processes: maintenance of methylation, de novo methylation and active DNA demethylation. CG and CHG methylation are maintained by methyltransferase 1 (MET1) and chromomethylase 3 (CMT3), while CHH methylation is preserved by chromomethylase 2 (CMT2) (Cao and Jacobsen 2002; Law and Jacobsen 2010). De novo methylation in all three sequence contexts is mediated through the RNA‐directed DNA methylation (RdDM) pathway, involving small interfering RNAs and a variety of proteins, including Dicer‐Likes (DCLs), Argonautes (AGOs), domains rearranged methyltransferases (DRMs) and other components like Defective in RdDMs (DRDs), RNA‐Dependent RNA Polymerases (RDRs), CLASSYs (CLSYs), Nuclear RNA Polymerase IVs (NRPDs) and Nuclear RNA Polymerase Vs (NRPEs) (Zhang et al. 2018). DNA demethylation is facilitated by the repressor of silencing 1 (ROS1)/demeter (DME) family of demethylases, including DME, ROS1, DME‐like 2 (DML2) and DML3 (Zhang et al. 2022).

While DNA methylation is well‐known for silencing transposable elements (TEs), its role in gene expression regulation is more complex. High levels of DNA methylation in promoters generally repress gene expression in many species (Zhang et al. 2018). Recent studies in peanuts have focused on deciphering DNA methylation patterns and their influence on traits such as disease resistance, oil content and stress responses (Bhat et al. 2020; Li, Li, et al. 2023; Liu et al. 2022; Wang et al. 2018). However, the relationship between DNA methylation and individual development under SS precision sowing compared with DS sowing remains unclear.

To explore this, we first examined growth differences between SS and DS under controlled indoor conditions. We used a DNA methylation inhibitor to assess the role of DNA methylation in these processes. By performing whole‐genome bisulfite sequencing (WGBS) and RNA sequencing (RNA‐seq), we obtained single‐base‐resolution DNA methylation profiles and gene expression patterns in leaves and roots from both SS and DS. Combined analyses of differential methylation, TE enrichment, and methylation‐related gene studies allowed us to investigate tissue‐specific epigenetic regulation in response to environmental stress. Integrating methylome, transcriptome and metagenomic data, we identified changes in DNA methylation patterns associated with planting patterns, shedding light on the epigenetic mechanisms that contribute to increased yield.

## 2 Results

### 2.1 Single‐Seed Precision Sowing Increased Peanut Growth

**FIGURE 1:**
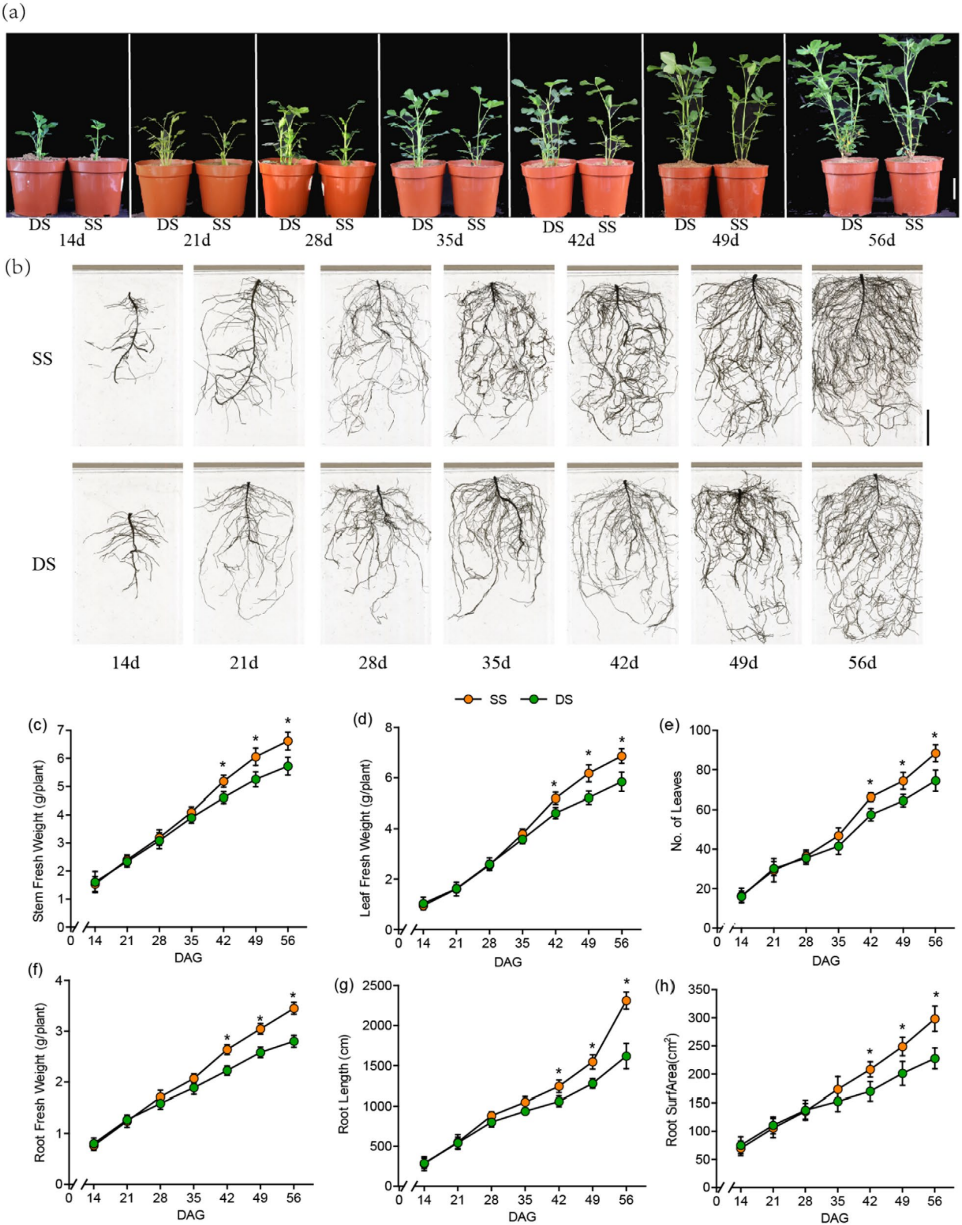
Growth comparison of peanuts under single seed (SS) and double seed (DS) conditions over time. (a) Phenotype of peanut shoots growth at different days after germination (14, 21, 28, 35, 42, 49, 56 DAG), (b) Phenotype of peanut root over the same periods. (c) stem fresh weight, (d) leaf fresh weight, (e) number of leaves, (f) root fresh weight, (g) root length, (h) root surface area. Bar = 5 cm. Data are presented as mean ± SD (c–e, *n* = 5 pots per treatment; f–h, *n* = 6–8 pots per treatment). Significant differences marked by asterisks between SS and DS in same time (*p* < 0.05, Student's *t*‐test).

In the field experiment, consistent with our previous work (Liang et al. 2020; Zhang et al. 2020), we found that SS precision sowing improved yield‐related metrics, including pod weight per plant, pod number per plant, full pod number per plant, kernel rate, biomass production per plant and 100‐pod weight (Table S1). Recognising that field experiments are subject to numerous external environmental influences, and given the susceptibility of DNA methylation to these factors, all subsequent experiments were conducted under controlled indoor greenhouse conditions (details in Materials and methods). First, we identified the periods when growth differences between SS and DS planting emerged under indoor conditions. We recorded the growth conditions of peanuts under the two planting patterns at 7‐day intervals. In shoots, as shown in Figure 1a, from 42 days after germination (DAG), the fresh and dry weights of stems and leaves in SS were significantly higher than in DS (Student's *t*‐test, *p* < 0.05; Figure 1c,d, Figure S1a,b), with a continuous increase observed at later times. The number of leaves also exhibited the same trend (Figure 1e). In roots, as shown in Figure 1b, similar to shoots, the fresh and dry weights differed starting from 42 DAG (Student's *t*‐test, *p* < 0.05; Figure 1f, Figure S1c). Additionally, root length and root surface area showed the same trend after calculation by WinRHIZO (Figure 1g,h). Overall, the results clearly demonstrate that SS significantly enhances both above‐ground and below‐ground plant growth compared to DS indoors from 42 DAG. Therefore, this time point was adopted in the subsequent experiment.

### 2.2 The Role of DNA Methylation in Different Planting Pattern

**FIGURE 2:**
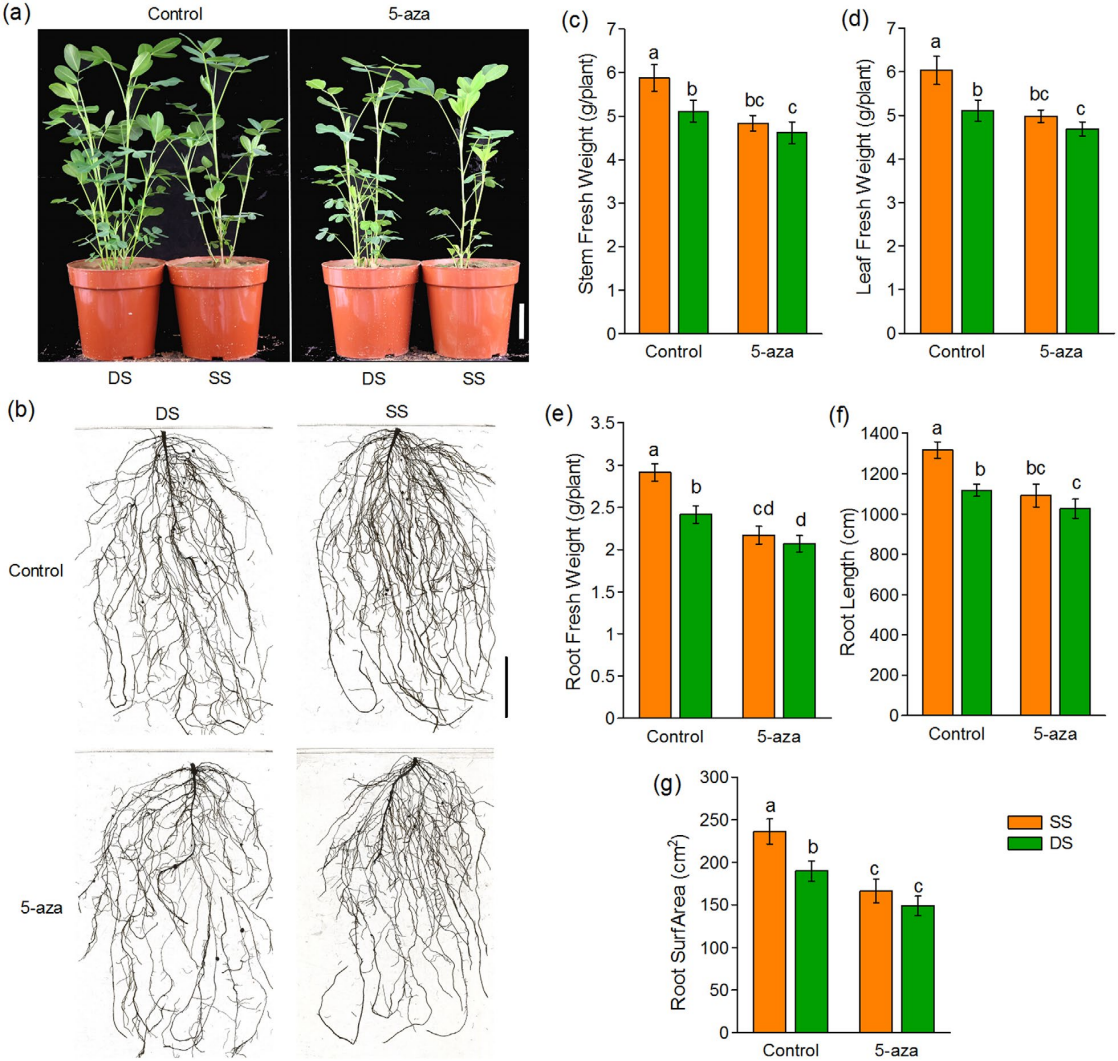
Impact of 5‐aza on peanut growth under SS and DS conditions. (a) Phenotypic alterations observed in peanuts under SS and DS conditions with or without 5‐aza sprayed, (b) phenotype of peanut roots, (c) stem fresh weight per plant, (d) leaf fresh weight per plant, (e) root fresh weight per plant, (f) root length and (g) root surface area. Bar = 5 cm. Data are presented as mean ± SD (a–c, *n* = 5 pots per treatment; d–g, *n* = 6–8 pots per treatment). Different letters above bars indicate significant differences (*p* < 0.05).

To test whether DNA methylation is involved in the increased peanut growth observed with single‐seed precision sowing, the DNA methyltransferase inhibitor 5‐azacytidine (5‐aza) was employed in this study (Goffin and Eisenhauer 2002; Griffin et al. 2016). According to the results in Figure 1, plants were sprayed with 5‐aza or water from 28 to 42 DAG, as growth differences emerged from 42 DAG. To minimise adverse effects on peanuts, we determined the optimal concentration of 5‐aza. Spraying 5‐aza at 20 μM significantly inhibited the fresh weight of stems, leaves and roots; these adverse effects increased with higher concentrations of 5‐aza, though little effect was observed at 10 μM (Figure S2). Therefore, we used 20 μM 5‐aza in this study. We compared the phenotypes of two planting patterns with and without 5‐aza to minimise differences in DNA methylation status. As shown in Figure 2a,b, similar to Figure 1, the fresh and dry weights of stems, leaves and roots in SS were higher than in DS under control conditions (Figure 2c–e, Figure S3). However, no significant weight differences were found between SS and DS in the 5‐aza treatment (Figure 2c–e). Root length and root surface area showed the same trend (Figure 2f,g). Notably, two‐way ANOVA of all measured values under control and 5‐aza conditions revealed a significant interaction between planting pattern and DNA methylation. This result suggests that DNA methylation status in peanut plants might be responsible for the growth increase observed with single‐seed precision sowing.

#### 2.2.1 Genome‐Wide Profile of DNA Methylation in Different Planting Pattern

To investigate the characteristic features and patterns of DNA methylation in response to planting patterns, we conducted single‐base resolution whole‐genome bisulfite sequencing (WGBS) on DNA extracted from peanut leaves and roots. We sequenced a total of 12 samples, comprising three biological replicates. Replicates produced an average of 316 million 150 bp reads each, with > 91.67% clean reads and > 97.45% bases at Q30 or higher. Bisulfite conversion efficiency was consistently > 99.54%. Moreover, > 78.26% of the reads were successfully mapped to the published Tifrunner ( *Arachis hypogaea* ) genome assembly (gnm2) (Table S2), indicating that the sequencing data were of sufficient quality and quantity for subsequent analysis.

**FIGURE 3:**
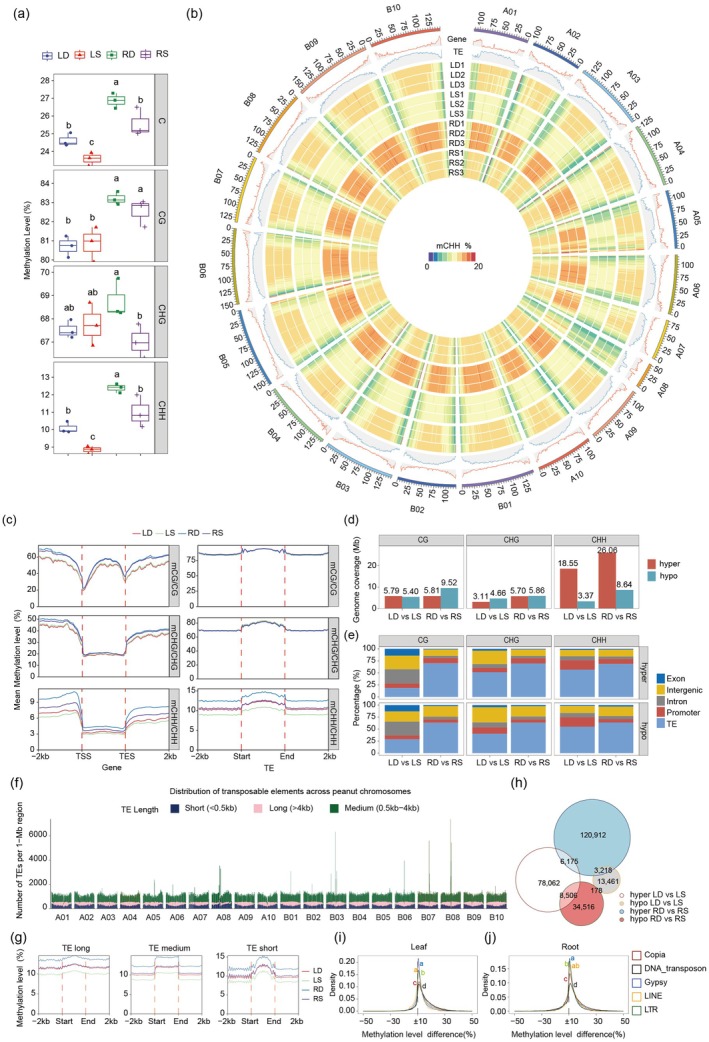
Characterisation of DNA methylation status in peanut leaves and roots under SS and DS conditions. (a) Cytosine methylation levels. LD, peanut leaf in DS; LS, peanut leaf in SS; RD, peanut root in DS; RS, peanut root in SS. Data are means ± SD (*n* = 3). Different letters above boxplots indicate statistically significant differences (*p* < 0.05). (b) Genome‐wide profiles of CHH DNA methylation across the peanut chromosomes. A 1‐Mb window was used for calculating the gene/repeat density or methylation levels. The colour scale represents the percentage of methylation levels. (c) Metaplots depict the mean CG, CHG and CHH methylation levels within each gene, transposable element (TE) and the 2 kb flanking regions. Each gene, TE and their flanking regions were evenly divided into 50 bins. (d) A stacked bar chart presents the genome coverage of differentially methylated regions (DMRs). (e) Proportion of DMRs at genomic regions (exon, intron, promoter, TE and intergenic) across three sequence contexts. (f) Distribution of TEs of varying lengths (short, medium, long) across peanut chromosomes. (g) Metaplots depict the mean CHH methylation levels of TEs of different lengths within each TE and the 2 kb flanking regions. (h) A Venn diagram illustrates the overlap of hyper‐ and hypomethylated CHH DMRs. (i, j) Density plots show the differences in CHH methylation levels across various TE families (*Copia*, DNA transposon, *Gypsy*, LINE, LTR) in leaves (i) and roots (j).

We first analysed the mean methylation level, which reflects the extent of methylation. The mean methylation levels were higher in DS than in SS patterns for both leaves and roots (Figure 3a). In terms of context, higher CHH methylation was observed in leaves, while higher CHG and CHH methylation were present in roots. We then examined the mean methylation density, which indicates the presence or absence of methylation. Consistent with Figure 3a, the mean methylation density was higher in DS than in SS for both leaves and roots (Figure S4), though only higher CHH methylation was observed. Additionally, a higher proportion of CHH methylation was found in DS compared with SS in both leaves and roots (Figure S5).

Both the methylation level and density analyses suggest that CHH methylation is more influenced by planting pattern. The circular heat map and violin plot further highlight the changes in CHH methylation levels due to planting pattern (Figure 3b, Figure S6), indicating that DS altered asymmetric DNA methylation, involved in de novo DNA methylation, and increased DNA methylation levels in peanut compared with SS.

To profile CHH methylation levels at the chromosome level, we divided the peanut genome into 1 Mb bins and calculated methylation levels, gene density and TE density in each bin. As consistently observed in previous studies (Bhatia et al. 2018; Springer et al. 2016; Williams et al. 2022), the densities of TEs and genes were negatively correlated, and the methylation levels of CHH were consistent with the density trend of TEs (Figure 3b). Furthermore, we analysed genes and TEs and examined their DNA methylation affected by planting pattern in leaves and roots. No significant variation in CG and CHG methylation in genes and TEs was observed between SS and DS in either leaves or roots (Figure 3c). However, CHH methylation levels at genes and TEs were significantly increased in both leaves and roots under DS compared to SS. These results were consistent with the observations of planting pattern influence on global DNA methylation patterns in leaves and roots (Figure 3a,b, Figures S4–S6).

#### 2.2.2 Characterisation of Differentially Methylated Region

To detect DNA methylation variations caused by different planting patterns, we identified differentially methylated regions (DMRs) under DS compared to SS in peanut leaves and roots (Figure 3d). In both tissues, methylation variations were primarily observed in the CHH context, leading to approximately 0.89% and 1.41% variation in the genome for leaves and roots, respectively. Only a limited number of DMRs were detected in the CG and CHG contexts. In leaves, there were abundant CHH hyper‐DMRs under DS compared with SS, accompanied by some hypo‐DMRs in the CHG context, while the number of DMRs in the CG context showed no evident trend. In roots, we also observed abundant CHH hyper‐DMRs under DS compared to SS, accompanied by some hypo‐DMRs in the CG context, while the number of DMRs in the CHG context showed no evident trend.

We also analysed the distribution of DMRs across the genome and found that the distribution of hyper‐DMRs and hypo‐DMRs was similar. In roots, more than 69% and 63% of hyper‐DMRs and hypo‐DMRs, respectively, were located in TE regions under DS compared to SS, followed by intergenic, promoter, intron and exon regions in all three contexts (Figure 3e). In leaves, the distribution of DMRs was context‐dependent. Although the distribution of CHG‐DMRs and CHH‐DMRs was similar to roots with smaller TE and larger intergenic regions, the CG context showed a different pattern. For hyper‐CG‐DMRs, the region order was intron, intergenic, TE, exon, and promoter, while for hypo‐CG‐DMRs, it was TE, intron, intergenic, exon and promoter. The differences in DMR distribution suggest distinct mechanisms for the effect of DNA methylation on planting patterns in different tissues.

#### 2.2.3 Distinct Regulatory Mechanisms of TE Methylation Patterns

Different CHH methylation patterns have been observed across various types of TEs (Ma et al. 2018; Wang et al. 2016). Since most DMRs were located on TEs, with hyper‐CHH methylation being the most evident change, we focused our analysis on TEs and CHH methylation. We classified the TEs into short (< 0.5 kb), medium (0.5–4 kb) and long (> 4 kb) categories as previously described (Wicker et al. 2007). Examination of the genomic distribution of these three TE types revealed that short and medium TEs exhibit nearly complementary patterns across the chromosomes (Figure 3f). In regions where short TEs are more abundant, medium TEs are less dense, and vice versa. Medium TEs are more uniformly distributed across the chromosomes, with some peaks in specific regions (e.g., on A08, B03 and B08), indicating areas of increased medium TE activity. The distribution of short TEs closely resembled that of genes (Figure 3b). The mean CHH methylation levels followed the pattern RD (peanut root in DS) > RS (peanut root in SS) ≈ LD (peanut leaf in DS) > LS (peanut leaf in SS) across upstream, body and downstream regions in all three TE classes (Figure 3g), similar to the pattern observed for TEs in Figure 3c. Although the comparisons of RD vs. RS and LD versus LS displayed similar trends, we further examined the consistency between the two tissues. There was little overlap in CHH‐DMRs between the two comparisons, indicating substantial differences between them (Figure 3h).

To further analyse the distribution of CHH hyper‐DMRs, we classified TE types in the peanut genome into five categories: Copia, Gypsy, LINE, LTR and DNA transposon, with the first four types belonging to Class I and the last one to Class II. Our results revealed that CHH hyper‐DMRs were highly enriched in Copia and DNA transposons in leaves (Kruskal–Wallis test, *p* < 0.05; Figure 3i, Table S3), as well as in DNA transposons in roots (Kruskal–Wallis test, *p* < 0.05; Figure 3j, Table S4). Despite this, the trend of mean CHH methylation levels was consistent across all TEs and the three TE classes in the five types investigated (Figure S7). The differences in TE types further underscore the distinct impact of planting patterns on DNA methylation in the two tissues.

#### 2.2.4 RdDM Pathway Accounting for the DNA Methylation Variations in Peanut Planting Patterns

**FIGURE 4:**
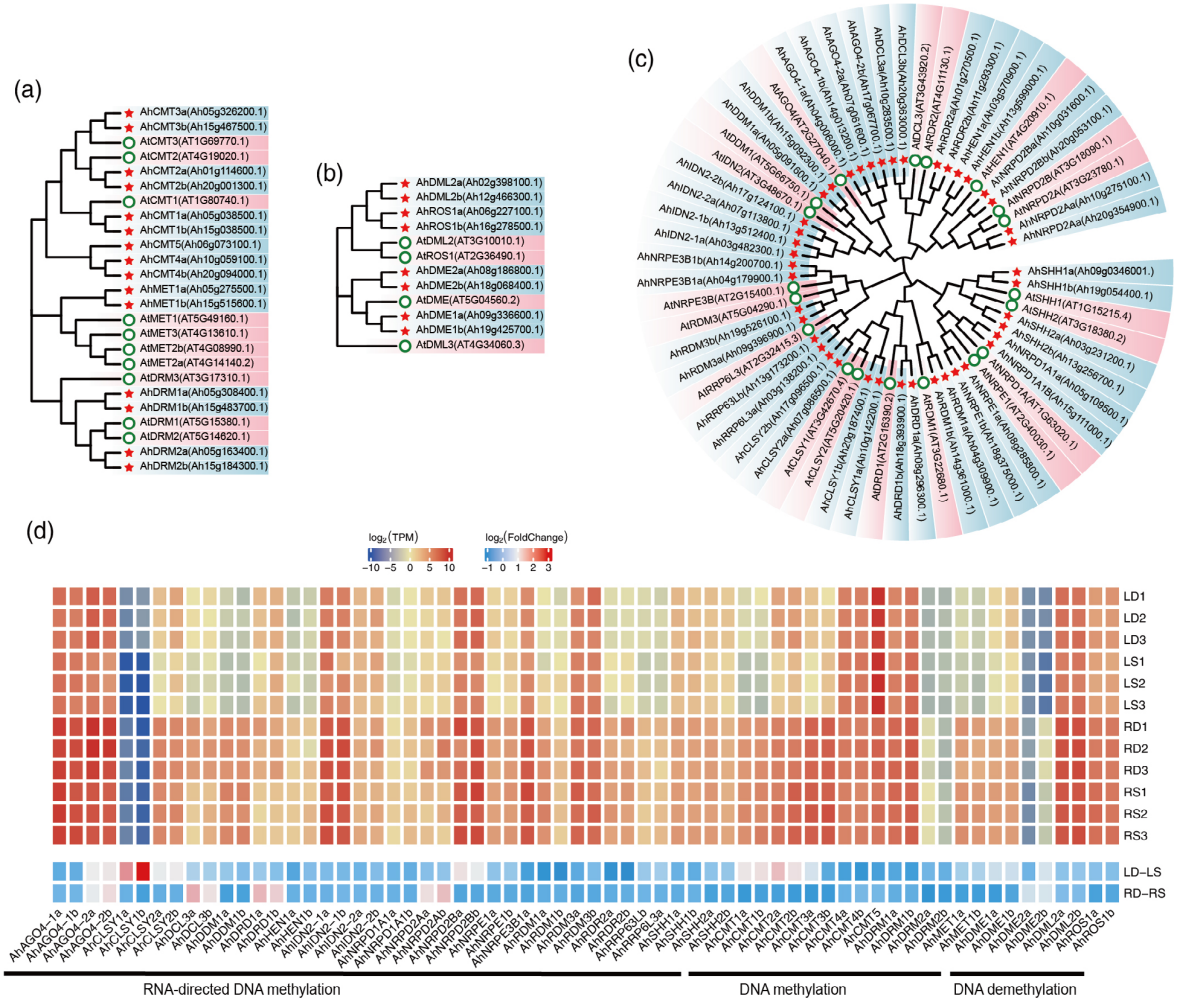
Phylogenetic and expression analyses of DNA methylation‐related genes. Phylogenetic analyses of DNA methylation (a), DNA demethylation (b) and RNA‐directed DNA methylation (c) genes in peanut (red star) and *Arabidopsis* (white circle). Neighbour‐joining phylogenetic trees were constructed in MEGA11 with 1000 bootstrap replicates and Poisson correction. (d) Heatmap showing transcript levels of DNA methylation‐related genes in peanut leaves and roots under SS and DS conditions. The value was shown after log_2_ transformed.

The global increase in methylation levels in DS compared to SS may be primarily attributable to increased cytosine methylation in the CHH context. Dynamic changes in DNA methylation are closely related to the expression patterns of DNA methyltransferase and demethylase genes, as well as the RNA‐directed DNA methylation (RdDM) pathway (Matzke and Mosher 2014). In this study, we identified 65 methylation‐related genes in the peanut reference genome, including 15 DNA methylation genes and 8 DNA demethylation genes through HMM search, as well as 42 RdDM genes through DIAMOND search (Figure 4a–c). As an allotetraploid (AABB, 2*n* = 4× = 40), almost all methylation‐related genes had a homologue in the subgenome, except for *AhCMT5*.

To examine the expression levels of these genes, RNA‐seq analyses were conducted on 12 libraries from peanut samples using the same treatment as WGBS, with three biological replicates. The raw reads obtained from RNA‐seq were trimmed, filtered and high‐quality reads were aligned to the peanut reference genome. Over 92.22% of the RNA‐seq reads were mapped to the reference genome, with at least 70.85% of the uniquely mapped reads used for further analysis (Table S5). Nearly all methylation‐related genes were detected, with the exception of *AhNRPE3B1b*, which was not expressed in either of the two tissues examined. In roots, all differentially expressed genes were associated with the RdDM pathway, including *AhAGO4‐2a*, *AhAGO4‐2b*, *AhDCL3a*, *AhDCL3b*, *AhDRD1a*, *AhDRD1b*, *AhNRPD2Aa* and *AhNRPD2Ab* in RD compared with RS (Figure 4d). In leaves, *AhCMT1a*, *AhCMT1b*, *AhCMT2a* and *AhCMT2b* were involved in DNA methylation, in addition to *AhAGO4‐2a*, *AhAGO4‐2b*, *AhCLSY1a*, *AhCLSY1b*, *AhCLSY2a*, *AhCLSY2b*, *AhNRPD2Ba* and *AhNRPD2Bb* in the RdDM pathway in LD compared with LS. All the mentioned genes were upregulated in DS compared with SS in both leaves and roots, contributing to the increased CHH methylation levels. The differences in methylation levels between the two tissues further suggest distinct mechanisms operating under different planting patterns.

The RdDM pathway is primarily mediated by 24‐nucleotide small interfering RNAs (siRNAs) (Matzke and Mosher 2014). To strengthen our analysis, we conducted small RNA sequencing on leaf and root samples under SS and DS conditions (Table S6). Consistent with the observed CHH methylation trends (Figure 3), we detected a higher abundance of 24‐nt siRNAs in DS compared with SS in both tissues. Specifically, 24‐nt siRNAs were significantly enriched in CHH hypermethylated regions, while their presence was reduced in CHH hypomethylated regions under both SS and DS conditions (Student's *t*‐test, *p* < 0.0001; Figure S8). These findings strongly support the role of 24‐nt siRNAs in driving CHH methylation increases, likely through the canonical RdDM pathway.

### 2.3 Relationship Between DNA Methylation and Gene Expression

To assess the correlation between DNA methylation and gene expression, we performed both WGBS and RNA‐seq analyses using leaf samples from three treatments: DS, SS and DS + 5‐aza (Figure S9). We categorised genes into four expression groups based on their transcript per million (TPM) values: none (TPM = 0), and low, moderate and high (equal divisions among genes with TPM > 0). Our analysis focused on CHH methylation, as it is highly dynamic and relevant to RdDM‐mediated regulation. As shown in Figure S9, genes with no expression exhibited the highest levels of CHH methylation across all treatments. For expressed genes, promoter regions consistently showed higher CHH methylation than gene bodies or downstream regions. This pattern held true regardless of treatment and aligns with previous findings in model and crop species such as *Arabidopsis*, *soybean* and *cassava* (Veley et al. 2023; Zhang et al. 2018). Importantly, promoter CHH methylation was negatively correlated with gene expression, particularly in lowly expressed genes. This negative correlation was most pronounced in the order: DS > SS > DS + 5‐aza. The reduction of promoter methylation in DS + 5‐aza plants was accompanied by a relative increase in gene expression, providing functional evidence that DNA methylation plays a repressive role in gene regulation.

#### 2.3.1 DS Enhancing WRKY‐Mediated Leaf Senescence

**FIGURE 5:**
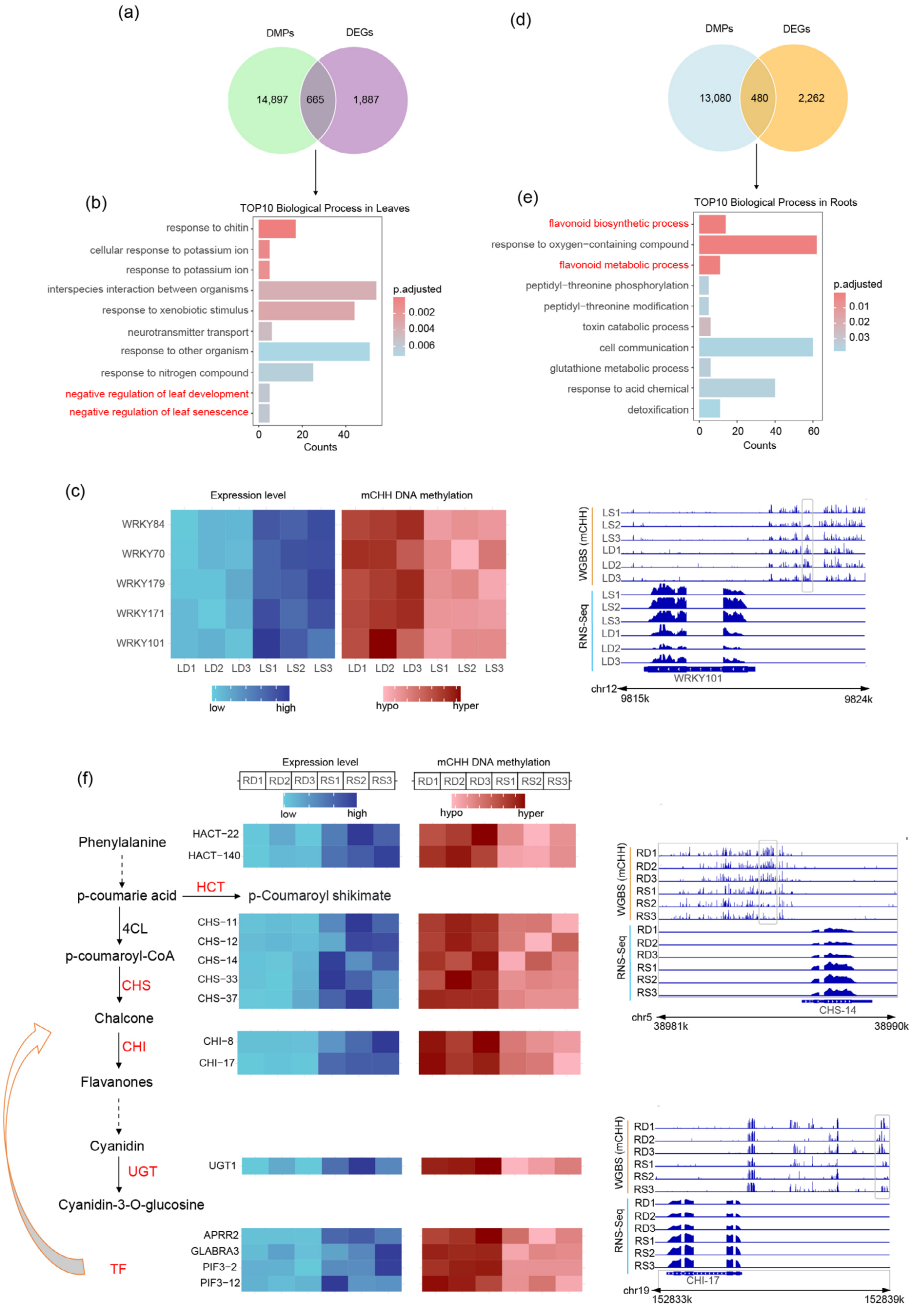
Conjoint analysis of leaves and roots transcriptomes and methylomes under two planting patterns. Venn diagrams show the overlap between downregulated differentially expressed genes (DEGs) and differentially methylated promoter (DMP)‐associated genes in leaves (a) and roots (d). The top 10 enriched biological processes in leaves (b) and roots (e) are presented. (c) The left panel (heatmap) displays the expression levels and DNA methylation levels in the promoters of WRKY genes. The right panel (genome browser snapshot) illustrates the DNA methylation and expression of the WRKY101 gene under LD and LS treatments. The grey box indicates the DMR. (f) The flavonoid metabolic pathway is shown. Enzymes marked in red indicate that their corresponding genes were differentially expressed and that their promoters exhibited differential methylation between RD and RS, as detailed in the heatmap. The right panel (genome browser snapshot) presents the DNA methylation and expression of the CHS‐14 and CHI‐17 genes under RD and RS treatments. The grey box indicates the DMR. The heatmap data in panels (c) and (f) were log_2_‐transformed and normalised.

Given that leaves and roots exhibit different mechanisms, we first explored the mechanism in leaves through a conjoint analysis of transcriptomes and methylomes. Methylation in promoter regions regulates gene expression, typically showing a negative correlation with gene expression levels (Figure S9). Since hyper‐CHH was the predominant DMR, we identified 15 562 differentially methylated promoters (DMPs) located within hyper‐CHH DMRs and 2552 downregulated differentially expressed genes (DEGs) between DS and SS, with 665 overlapping genes (Figure 5a).

To understand the functions of the overlapping genes, we performed gene ontology (GO) enrichment analysis to identify enriched biological processes. We found that ‘negative regulation of leaf senescence’ and ‘negative regulation of leaf development’ were among the top 10 enriched biological processes, both associated with leaf weight and number (Figure 5b, Table S7). These two GO terms shared five genes, all of which belonged to the WRKY transcription factor family (Table S8). Members of this family are characterised by the highly conserved WRKY domain, whose prominent features include the conserved WRKYGQK sequence and zinc finger motifs (C_2_H_2_ or C_2_HC) (Javed and Gao 2023). The heatmap and genome browser snapshot were confirmed the hyper‐CHH methylation in promoter and downregulated express level (Figure 5c). To further confirm the role of WRKY, we measured the content of chlorophyll a and chlorophyll b, which are involved in leaf senescence. Compared with SS, DS exhibited lower chlorophyll content (Figure S10), that suggests that the downregulation of WRKY genes may contribute to accelerated leaf senescence and impaired leaf development. These findings highlight the critical role of DNA methylation, particularly in promoter regions, in regulating gene expression in response to different planting patterns. SS enhancing flavonoid‐mediated nodule development in root.

A similar analysis was conducted in roots, where we identified 13 560 DMPs and 2742 DEGs, with 480 overlapping genes between them (Figure 5d). The most enriched GO term was the flavonoid metabolic process, closely followed by the flavonoid biosynthetic process (Figure 5e, Table S9). These GO categories included 14 and 11 genes, respectively, with all 11 genes in the flavonoid biosynthetic process also being part of the flavonoid metabolic process. The flavonoid metabolic process involved five chalcone synthases (CHSs), two chalcone‐flavanone isomerases (CHIs), one glycosyltransferase (UGT), two acyl‐transferases (HATs) and four transcription factors (TFs) (Figure 5f). The heatmap and genome browser snapshot revealed decreased CHH methylation levels in the promoters and increased expression levels of these 14 genes in SS compared with DS.

**FIGURE 6:**
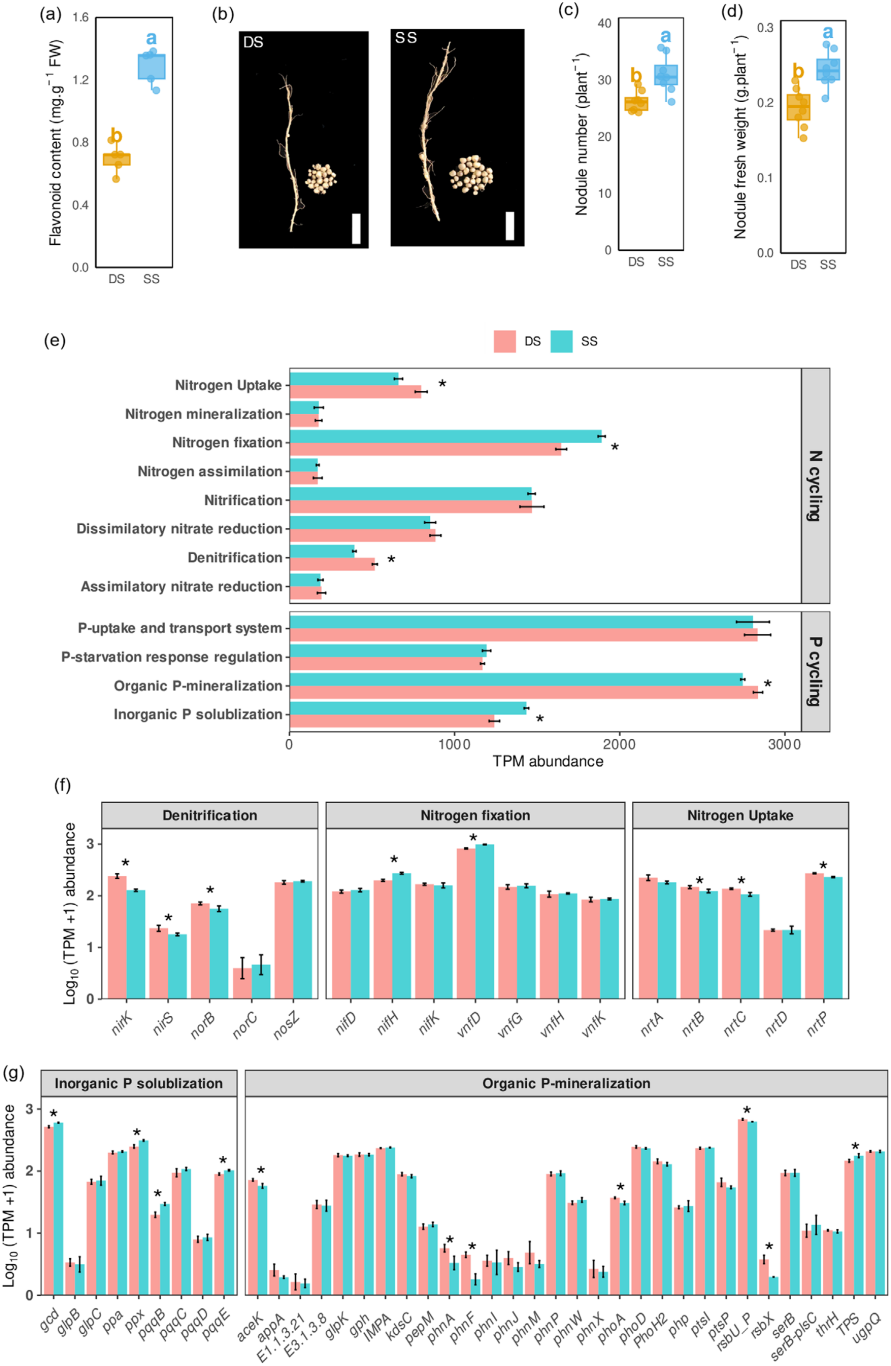
Functional profiles of rhizosphere microbiomes based on metagenomic sequencing. (a) Flavonoid content, (b) Peanut nodulation condition, bar = 1 cm, (c) nodule number, (d) nodule fresh weight, (e) gene transcripts with zero transcripts per million (TPM) abundance related to N and P cycling processes based on metagenomic sequencing under DS and SS treatments. The TPM abundance of N cycling selected genes (f) and P cycling selected genes (g) was log_10_‐transformed. (a) *n* = 5, (c, d) *n* = 8, (e–g) *n* = 3. Different letters or asterisk indicate significant differences between SS and DS treatments (*p* < 0.05, Student *t*‐test).

Subsequently, we analysed the flavonoid content under the two planting patterns. As shown in Figure 6a, there was an 84.84% increase in flavonoid content in SS compared with DS. Flavonoids are known to enhance antioxidant capacity and stress resistance in various plants. In our study, SS also led to a significant reduction in malondialdehyde (MDA) accumulation, as well as a decrease in hydrogen peroxide (H_2_O_2_) and superoxide anion (O_2_·^−^) production by 32.67%, 39.11% and 46.54%, respectively, compared with DS (Figure S11). The activities of superoxide dismutase (SOD), peroxidase (POD) and catalase (CAT) were 32.34%, 42.57% and 63.40% higher in DS than in SS, respectively (Figure S11). The formation of root nodules in leguminous plants is initiated by the secretion of flavonoids from the plant roots, which are sensed by rhizobia (Desbrosses and Stougaard 2011). We further investigated the condition of root nodules and found that the number of pods per plant increased by 18.06% under SS compared to DS, with the pod weight per plant increasing by 26.32% accordingly (Figure 6b–d). Additionally, the total N and P content in peanut roots grown in pots increased significantly by 26.09% and 18.75%, respectively, under SS compared with DS (Figure S12). However, K content remained unaffected by the planting pattern. Analysis of the rhizosphere soil physicochemical properties revealed that DS reduced ammonium‐N, nitrate‐N and available P contents by 19.90%, 46.12% and 10.26%, respectively, compared with SS. Notably, soil organic matter content also decreased significantly by 8.61% in DS compared with SS (Table S10). This suggests that soil microbes may influence the macroelement changes associated with the induction of root nodules.

#### 2.3.2 Metagenomic Analyses in Flavonoid‐Induced Nodule Development

To further confirm the role of flavonoid‐induced nodule development, metagenomic sequencing was conducted on six soil samples from the rhizosphere under SS and DS treatments. After quality control assessment, the mean numbers of clean reads and contigs were 80 104 685 and 371 828, respectively (Table S11). A total of 6681 KEGG orthology identifiers (KOs) were obtained from all samples. Given that ammonium‐N, nitrate‐N and available P were influenced by the planting pattern, the analysis focused on soil N and P cycling. In total, 38 soil N cycle genes and 65 soil P cycle genes, along with their corresponding KO numbers, were identified based on previous studies (Bigatton et al. 2024; Liu, Gao, et al. 2023; Ma et al. 2020). These genes were classified into six and four pathways according to their functions in the soil N and P cycles, respectively (Table S12). As shown in Figure 6e, the denitrification, nitrogen fixation and nitrogen uptake pathways in N cycling, as well as inorganic phosphorus solubilisation and organic phosphorus mineralisation in P cycling, were significantly influenced by the planting pattern. Specifically, SS increased nitrogen fixation and inorganic phosphorus solubilisation while decreasing denitrification, nitrogen uptake and organic phosphorus mineralisation. In N cycling, 60% of the genes in both the denitrification and nitrogen uptake pathways were reduced by SS compared with DS (Figure 6f), while only two genes (*nifH* and *vnfD*) were upregulated in the nitrogen fixation pathway, accounting for 28.57%, implying that the decreases in the two pathways played a more significant role. In P cycling, the inorganic phosphorus solubilisation pathway is more critical than the organic phosphorus mineralisation pathway, with 44.44% and 23.33% of genes being influenced by the planting pattern, respectively (Figure 6g). The metagenomic analysis revealed significant shifts in N and P cycling processes due to different planting patterns and underscored the critical role of microbial communities in nutrient cycling and their potential impact on plant growth.

## 3 Discussion

### 3.1 Epigenetic Mechanisms Underlying Competitive Exclusion in SS Precision Sowing of Peanut

**FIGURE 7:**
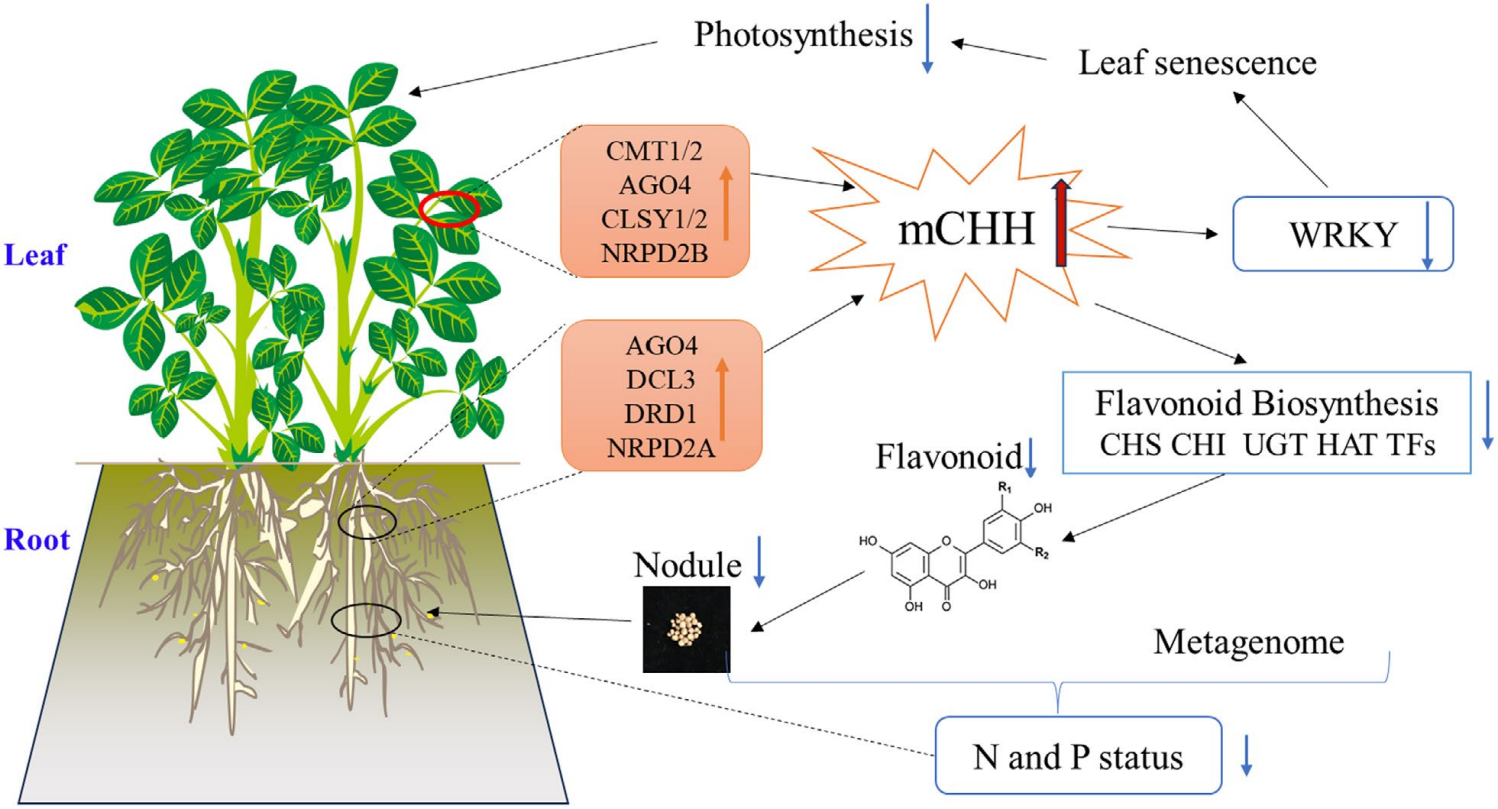
Hypothetical model of the epigenetic mechanism for single‐seed precision sowing increased peanut yield.

Competitive exclusion, where plants vie for limited resources like light, water and nutrients, often impairs growth and yield, especially under stress (Hardin 1960). Our previous peanut studies showed that SS precision sowing enhances yield over DS sowing by optimising resource use and reducing competition (Liang et al. 2020; Yang et al. 2023, 2021; Zhang et al. 2020). However, field‐based results were confounded by environmental variability (Rezaei et al. 2023). To isolate the specific effects of sowing methodology, we conducted greenhouse experiments controlling key environmental factors—water, light, temperature, nutrients, soil and seed source. Despite identical genetic backgrounds and these controlled conditions, transcriptome analysis revealed numerous DEGs between SS and DS plants (Figure S13), unlike fewer DEGs in field studies (Yang et al. 2021), suggesting regulatory mechanisms beyond genetics or direct environmental cues. Epigenetic regulation, particularly DNA methylation, is a well‐established mechanism mediating plant adaptive responses to environmental stimuli (Chinnusamy and Zhu 2009). For instance, in maize, planting density influences yield via methylation levels, with lower methylation linked to higher gene expression and yield under high‐density conditions (Peng and Zhang 2009; Tani et al. 2005). We therefore hypothesised that differential DNA methylation patterns drive the observed growth and developmental differences between SS and DS peanut plants. Application of 5‐aza, a demethylating agent, abolished the phenotypic advantages previously observed in SS plants (Figure 2, Figure S3), providing strong evidence for the pivotal role of DNA methylation in planting patterns. Consistent with this, DS plants exhibited hypermethylation at many growth‐related loci compared with SS plants (Figure 7). Further supporting our hypothesis, 5‐aza treatment led to a significant decrease in promoter CHH methylation levels in DS plants (Figure S9). This reduction in methylation was associated with an increase in the expression of corresponding genes, likely including these growth‐related loci. Such a negative relationship, where higher promoter methylation levels are linked to suppressed gene activity (and vice versa), is a well‐documented principle in epigenetic regulation across diverse model and crop species (Zhang et al. 2018). However, it is important to clarify that 5‐aza typically causes a global reduction in methylation levels (Griffin et al. 2016; Liu et al. 2022). Notably, the application of 5‐aza results in a more pronounced reduction in growth for SS plants relative to DS plants (Figure 2), highlighting the role of DS‐specific methylation in growth modulation. This study innovatively integrates epigenetic considerations into the framework of competitive exclusion research, thereby extending both ecological and molecular insights (Yang et al. 2023; Zhang et al. 2020). Furthermore, it aligns with the growing interest in epigenetic approaches for crop optimisation (Gallusci et al. 2017; Zhang and Zhu 2025). It provides a novel framework for understanding plant responses to cultivation practices, with potential applications in precision agriculture.

### 3.2 CHH Methylation and siRNA‐Directed Regulation of Peanut Development

CHH methylation is recognised as a key epigenetic mechanism influencing gene expression and phenotypic adaptation, particularly in response to agricultural practices (Zhang and Zhu 2025). Our WGBS analysis revealed that DS sowing induces elevated CHH methylation in peanut leaves and roots compared with SS precision sowing (Figure 3). The observed CHH hypermethylation in DS planting is likely driven by a combination of physical interaction and chemical signalling. Physical contact—such as root intertwining or shoot competition—can induce mechanical stress, potentially activating the RdDM pathway. This is supported by the accumulation of 24‐nt siRNAs in DS (Figure S8), in line with previous reports on stress‐induced epigenetic responses (Wibowo et al. 2016). In parallel, chemical cues—possibly from altered root exudates or intensified nutrient competition—may further stimulate RdDM activity. This is corroborated by the siRNA enrichment and known roles of chemical stress in modulating methylation (Zhang et al. 2018). Consistent with this, our RNA‐seq data reveal the upregulation of key RdDM components (e.g., *AhAGO4‐2a*, *AhDCL3a*) in DS (Figure 4d), suggesting that both physical and chemical signals converge to reinforce CHH hypermethylation through the RdDM pathway.

SS precision sowing, with lower CHH methylation, enhanced flavonoid production, increasing nodule numbers and nutrient uptake, as evidenced by higher nitrogen and phosphorus content (Figure 6, Figure S12). This aligns with studies showing methylation‐mediated regulation of secondary metabolism in legumes (Gupta et al. 2019). Conjoint methylome and transcriptome analyses confirmed a negative correlation between promoter methylation and gene expression, particularly for flavonoid pathways, consistent with findings in apple and tea (Chen et al. 2025; Xu et al. 2024). The identification of two acyltransferases (HATs) and four transcription factors, potentially regulated by methylation like *bHLH39* in other species (Zhu et al. 2023), suggests a novel mechanism in flavonoid biosynthesis (Shen et al. 2022).

Consistent with previous studies, distinct tissue‐specific methylation responses were observed (Bhatia et al. 2018; Williams et al. 2022). In leaves, *AhCLSY1a/b* and *AhCLSY2a/b* upregulation in DS plants directed CHH methylation of *AhWRKY* gene promoters, phylogenetically related to Arabidopsis *WRKY54* and *WRKY70* (Besseau et al. 2012). This downregulated *AhWRKY* expression, accelerating leaf senescence and reducing photosynthesis (Figure 5). In roots, CHH hypermethylation suppressed flavonoid biosynthesis genes (*CHS*, *CHI*, *UGT*) and associated transcription factors (Figures 5 and 6), impairing nodule development critical for nitrogen fixation (Desbrosses and Stougaard 2011). In TEs, DNA transposons were predominantly hypermethylated in roots, whereas both *Copia* retrotransposons and DNA transposons exhibited hypermethylation in leaves (Figure 3). This suggests active siRNA‐directed silencing of these genomic elements, a known defence mechanism (Bhatia et al. 2018; Widman et al. 2014). Unlike cotton, where short TEs peak in gene bodies (Ma et al. 2018), medium TEs in peanuts showed elevated methylation across entire gene bodies, highlighting species‐specific TE regulation. These findings elucidate how CHH methylation and siRNA pathways orchestrate peanut development, offering novel insights into epigenetic optimisation of legume cultivation.

### 3.3 Metagenomic Insights Into Nutrient Cycling and Epigenetic Regulation in SS Precision Sowing

Flavonoids, key signalling molecules in plant–rhizobia symbiosis, initiate nodulation by inducing rhizobial *nod* gene expression, a process critical for nitrogen fixation in legumes like peanut (Desbrosses and Stougaard 2011). SS precision sowing enhances pod number and weight, suggesting improved nodulation and nitrogen fixation compared with DS sowing (Figure 6). This is supported by increased N and P content in SS roots, driven by flavonoid‐mediated nodule formation and enhanced mycorrhizal associations (Bona et al. 2017). Epigenetic regulation, particularly reduced CHH methylation in SS roots, promotes flavonoid biosynthesis genes (*CHS*, *CHI*, *UGT*), facilitating these symbiotic interactions (Zhang et al. 2022). Metagenomic analysis revealed that SS sowing promotes soil microbial communities enriched in nitrogen fixation pathways, thereby contributing to enhanced nodulation and increased nitrogen content in plants (Figure 6). In contrast, DS soils exhibited elevated ammonium‐N and nitrate‐N levels, indicative of reduced microbial nitrogen assimilation and greater N losses via denitrification (Ravelo‐Ortega et al. 2023). For phosphorus, SS improved inorganic P solubilisation, likely through the increased abundance of phosphate‐solubilising bacteria, thus boosting P bioavailability (Richardson and Simpson 2011). Conversely, DS soils showed reduced organic P mineralisation and accumulated organic matter, collectively constraining P mobilisation. These findings align with studies on legume‐based systems, such as peanut–maize intercropping, where microbial nutrient cycling boosts yields (Dong et al. 2024; Qiao et al. 2024). Epigenetic mechanisms underpin these microbial interactions. In *Medicago truncatula* , DNA demethylation regulates nodule organogenesis, modulated by chromatin accessibility and histone modifications (Nagymihály et al. 2017; Satgé et al. 2016). In peanuts, hypomethylation in SS roots likely orchestrates gene expression for nodulation, integrating with microbial signals (Kumar and Wigge 2010; Zhang and Ogas 2009). Future research should investigate dynamic methylation changes during rhizobial infection and their interactions with histone modifications or Rhizobium signals. Identifying specific microbial taxa and functional genes driving N and P cycling will further elucidate these processes. Such insights could inform epigenetic marker‐based breeding strategies, enhancing legume productivity and agricultural sustainability (Dalakouras and Vlachostergios 2021).

In conclusion, we elucidate the epigenetic mechanisms by which SS precision sowing increases peanut yield. In leaves, DS sowing induces the expression of *CMT1/2*, *AGO4*, *CLSY1/2* and *NRPD2B*, leading to an increase in CHH methylation levels. Hyper‐CHH methylation in the promoters of *WRKY* genes downregulates their expression, which accelerates leaf senescence, reduces photosynthesis and ultimately decreases growth. In roots, DS similarly induces *AGO4*, *DCL3*, *DRD1* and *NRPD2A*, leading to elevated CHH methylation levels. The hyper‐CHH methylation of flavonoid biosynthesis gene promoters inhibits *CHS*, *CHI*, *UGT*, *HAT* and related transcription factors, which reduces flavonoid content and impairs nodule development. This decline in nodule number contributes to decreased nitrogen availability in the soil rhizosphere due to increased denitrification and reduced nitrogen fixation, as well as diminished phosphorus availability from increased inorganic P solubilisation and reduced organic P mineralisation, ultimately resulting in lower root nitrogen and phosphorus levels and reduced growth (Figure 7). Understanding these epigenetic mechanisms provides valuable insights for optimising agricultural practices and enhancing crop performance under varying environmental conditions.

## 4 Materials and Methods

### 4.1 Plant Materials and Growth Conditions

The study utilised the widely cultivated peanut cultivar ‘Huayu 22’ from Shandong Province, China. Soil samples were collected from the Yinmaquan experimental field of the Shandong Academy of Agricultural Sciences, located at 36°71′ N, 117°08′ E. The soil had a pH of 7.93, with an organic matter content of 11.83 g · kg^−1^, total nitrogen of 0.70 g · kg^−1^, available nitrogen of 88.63 mg · kg^−1^, total phosphorus of 0.62 g · kg^−1^, available phosphorus of 32.76 mg · kg^−1^, total potassium of 16.29 g · kg^−1^ and available potassium of 162.15 mg · kg^−1^.

For field experiment, single‐seed sowing treatment (one seed per hole, SS) consisted of 270 000 holes per hectare, while double‐seed sowing (two seeds per hole, DS) consisted of 135 000 holes per hectare; the distances between two adjacent holes were 10.0 cm and 18.5 cm in the SS and DS treatments, respectively. At harvest time, 10 peanut plants were randomly harvested and dried to determine the yield‐related metrics.

For indoor experiments, sieved and thoroughly homogenised soil, collected from a field site identical to that of the related field experiment and without a history of peanut cultivation, was used for pot experiments conducted under greenhouse conditions. The peanut seeds were surface‐sterilised with 70% (v/v) ethanol for 5 min, washed with sterilised ddH_2_O and germinated in a sterilised nutrient soil mixture containing 30% vermiculite. Seven‐day‐old seedlings were transplanted into plastic pots (14.5 cm diameter and 13 cm height), with one (single seed, SS) or two (double seed, DS) seedlings per pot containing 2 kg of soil. The pots were randomly arranged in the greenhouse, and the seedlings were grown under a light intensity of 6000 lx with a 16/8‐h light/dark cycle at 25°C/21°C (day/night). The soil moisture level was maintained at 60% of the field capacity, and a full nutrient supply was ensured using Hoagland nutrient solution. At 7‐day intervals, we recorded the growth conditions of peanuts. The fresh leaves and roots were collected for measuring weight; then the tissues were dried for testing dry weight. The fresh roots were washed clean for analysis by WinRHIZO (v 2019a). At 42 DAG, the top third leaf and whole root systems were collected for WGBS, RNA‐seq and small RNA sequencing. The collected leaf and root samples were immediately flash‐frozen in liquid nitrogen in the field and stored at −80°C until DNA or RNA extraction. For metagenome sequencing, rhizosphere soils were obtained by gently shaking off loosely attached bulk soil and collecting the soil adhering to the root surface. Soil samples were placed in sterile 2 mL tubes, transported on dry ice and stored at −80°C until further use. Genomic DNA and total RNA from plant tissues were extracted using standard CTAB and TRIzol protocols, respectively, with additional RNase and DNase treatments to ensure high‐quality nucleic acids. Soil DNA was extracted from 0.5 g rhizosphere soil samples using a MagPure Soil DNA LQ Kit (Magen Biotechnology, Guangzhou, China) following the manufacturer's protocols. DNA and RNA quality were assessed using agarose gel electrophoresis and Qubit fluorometry. Each type of sequencing was performed in triplicate by OE Biotech Co. Ltd. (Shanghai, China). For the 5‐aza treatment, plants were sprayed with 5‐aza (Sigma, St. Louis, MO, USA), freshly dissolved in sterilised double‐distilled water (ddH_2_O), from 28 to 42 DAG at different concentrations. The 5‐aza solution was freshly prepared each day to maintain chemical stability and efficacy. Regarding the application regimen, 5‐aza was sprayed once daily at a concentration of 20 μM for 14 consecutive days. Each plant received approximately 25 mL per application. An equal volume of ddH_2_O was used as a control treatment.

### 4.2 Whole‐Genome Bisulfite Sequencing and Data Analysis

For the WGBS library construction, the DNA was fragmented to 200–400 bp using a Covaris S220 sonicator, followed by blunt ending, 3′‐end dA tailing, and adaptor ligation with methylated adaptors to protect against bisulfite conversion. The ligated DNA was then bisulfite converted using the EZ DNA Methylation‐Gold kit. Different insert size fragments were excised from the same lane of a 2% tris‐acetate‐EDTA (TAE) agarose gel, purified using the QIAquick Gel Extraction Kit and amplified by PCR before Illumina sequencing. Raw sequencing data underwent preprocessing with fastp (v0.23.1, https://github.com/OpenGene/fastp), whereby reads were removed if they contained adapter sequences and low‐quality reads (quality < 20). The clean reads were then aligned to the cultivated peanut reference genome (gnm2.J5K5, https://www.peanutbase.org/data/public/Arachis_hypogaea/) using Bismark (v0.24.0) (Krueger and Andrews 2011) by the parameters ‐X 700 ‐score_min L, 0, −0.2. After deduplication using the deduplicate_bismark and methylation extraction using the bismark_methylation_extractor implemented in Bismark, the methylation state of every cytosine site was generated per treatment. To ensure accurate and reliable methylation calling, positions covered by at least four reads (≥ 4) were classified as low‐confidence, and their methylation ratios were omitted (Li, Liu, et al. 2023; Lowe et al. 2022; Yu et al. 2024). To address the potential impact of sequence mutations (SNPs) on methylation calling, we analysed SNP effects on CG, CHG and CHH methylation sites across 12 treatments using our whole genome DNA resequencing data and WGBS data. As detailed in Figure S15 and Table S13, SNPs have a negligible impact: of the mean 75.8 million CG sites, 94.9 million CHG sites and 232.4 million CHH sites, only 0.05%–0.07% are affected by SNPs, and 0.008%–0.05% are disrupted (e.g., by C > T or G > A mutations). These proportions, consistently low across treatments, indicate that SNP‐related effects are unlikely to confound our DMR analysis, which focuses on broad epigenetic patterns. Genome‐wide DNA methylation profiles were generated by calculating the methylation level as the ratio of methylated reads (#mC) to the total number of reads, both methylated and unmethylated (#mC + #umC). The methylation density was calculated as the ratio of methylated reads (#C) to the total reads (#C + #T). Each methylated read at a cytosine was counted as 1, while each unmethylated read was counted as 0. The statistical significance of differences in methylation levels between groups was assessed using Student's *t*‐test in R (v 4.2.1.), based on three biological replicates. Differentially methylated regions (DMRs) were identified using a sliding window approach (200 bp windows, 200 bp steps) implemented in the R package *methylKit* (v1.24.0) (Akalin et al. 2012). Significant DMRs were filtered based on a *q*‐value < 0.01 and context‐specific methylation difference thresholds (> 35%, > 25% and > 10% for CG, CHG and CHH contexts, respectively). The above criteria aligned with previously reported studies (He et al. 2024; Wu et al. 2024), effectively capturing biologically relevant methylation changes in our experimental context. This balance of sensitivity and specificity ensures robust detection of DMRs without excluding meaningful epigenetic signals. The overlap of DMRs with genomic features, such as transposons and genes, was calculated using BEDTools v2.30.0 (Quinlan and Hall 2010). The 2 kb upstream region of genes was defined as the promoter, and DMRs located within these promoter regions were extracted. Genes overlapping with these promoter‐associated DMRs were designated as differentially methylated promoters (DMPs).

To visualise the distribution of CHH methylation levels across treatments, violin plots were generated using the ggplot2 package in R. Each treatment group included three biological replicates, and methylation levels were calculated for 2551 nonoverlapping 1‐Mb windows per replicate. This provided sufficient data density to support distribution‐based visualisation. Statistical differences among treatments were assessed using Duncan's multiple range test, and groups with different letters were considered significantly different (*p* < 0.05). The violin plots were used in combination with circos plots to integrate both quantitative and spatial perspectives of methylation variation.

Metaplots of DNA methylation levels for genes and transposable elements (TEs) were generated using our local python3 script. Gene and TE body regions were proportionally divided into 50 bins, with upstream and downstream 2 kb regions divided into 50 bins of 40 bp. The CG, CHG and CHH methylation contexts were calculated and plotted separately.

### 4.3 RNA Sequencing and Data Analysis

Total RNA was extracted using TRIzol Reagent (Invitrogen, CA, USA) following the manufacturer's protocol. The cDNA library was prepared with the VAHTS Universal V6 RNA‐seq Library Prep Kit as per the manufacturer's recommendations. Sequencing of the cDNA library was performed using a paired‐end method on an Illumina Novaseq 6000, following the manufacturer's instructions. Adaptors and poor‐quality reads were removed using fastp (v0.23.1). High‐quality paired‐end reads were then aligned to the cultivated peanut reference genome using the Hisat2 alignment program (Pertea et al. 2016), and transcript abundance was calculated with RSEM (v1.2.25). Transcripts with zero TPM values were excluded from the analysis based on RSEM parameters. Raw read count data were imported into the R environment, combined into a data table containing fragment counts for each transcript, and a matrix was formed to identify DEGs using the R package edgeR (v3.40.2).

### 4.4 Small RNA Sequencing

For small RNA library construction, 1 μg of total RNA from each sample was processed using the NEBNext Small RNA Library Prep Set for Illumina (Cat. No. NEB#E7330S, New England Biolabs, USA) according to the manufacturer's protocol. The resulting libraries were sequenced on the Illumina NovaSeq 6000 platform, generating 150 bp paired‐end reads. Raw sequencing data underwent quality control filtering to remove low‐quality reads (Table S13), retaining only high‐quality clean reads ranging from 18 to 29 nucleotides in length. Clean reads were subsequently mapped to the cultivated peanut reference genome using ShortStack v4.0.3 with parameters ‐mmap f ‐pad 100 (Axtell 2013). The abundance of 24‐nt siRNAs was quantified by normalising mapped read counts to the total number of clean reads. Integration analysis of CHH DMRs and 24‐nt siRNA abundance was performed using BEDTools v2.30.0 (Quinlan and Hall 2010).

### 4.5 Identification of DNA Methylation‐Related Genes

Protein sequences of *Arabidopsis thaliana* DNA methylation‐related genes were obtained from the Arabidopsis genomic database (https://www.arabidopsis.org). The Hidden Markov Model (HMM) profiles were downloaded from the Pfam database in InterPro (https://www.ebi.ac.uk/interpro/). For DNA methylation maintenance genes, the PF00145 profile was used to search the peanut protein genome using the HMMER v3.3.2 program. DNA demethylation genes were identified using PF00730 and PF15628 profiles. For RNA‐directed DNA methylation genes, *Arabidopsis* protein sequences were used as seed sequences to search the peanut protein genome using DIAMOND (v2.1.9) (Buchfink et al. 2015). The best hits for homologous genes in the peanut plant were selected as candidates.

Multiple sequence alignments of DNA methylation‐related proteins from peanut and *Arabidopsis* were performed using the MUSCLE program with default parameters implemented in MEGA11 (Tamura et al. 2021). A phylogenetic tree was constructed in MEGA11 using the neighbour‐joining method, with 1000 bootstrap replicates and the Poisson correction method, while all other parameters were set to default. The phylogenetic tree was further displayed and edited using Evolview v3 (https://evolgenius.info/evolview/) (Subramanian et al. 2019).

### 4.6 Gene Ontology Analysis

All peanut proteins were submitted to eggNOG‐mapper (Cantalapiedra et al. 2021) to build Gene Ontology (GO) annotation reference. GO enrichment analysis was implemented using TBtools (v 2.112) (Chen et al. 2023). Genes with both *p* value < 0.01 and *q* value < 0.05 were considered to be significantly enriched.

### 4.7 Quantitative Real‐Time PCR (qRT‐PCR) Verification Analysis

Six methylation‐related genes were randomly selected to verify the accuracy of the omics data. cDNA synthesis, qRT‐PCR, primer design and calculations were performed according to our previous methods (Fan et al. 2023). All samples were analysed in three biological replicates. Detailed information on the specific primers and verification results can be found in Table S14 and Figure S16.

### 4.8 Metagenome Sequencing and Data Analysis

DNA was extracted from 0.5 g rhizosphere soil samples using a MagPure Soil DNA LQ Kit (Magen Biotechnology, Guangzhou, China) following the manufacturer's protocols. The quality of the extracted DNA was assessed by 1% agarose gel electrophoresis, while the concentration and purity were quantified using a NanoDrop 2000 UV–vis spectrophotometer (Thermo Scientific, Wilmington, NC, USA). All DNA samples were sequenced on the Illumina NovaSeq 6000 instrument. Reads were trimmed and filtered using fastp (v0.23.1), and the postfiltered paired‐end reads were aligned to the host genome using Bowtie2 (v2.5.1) (Langmead and Salzberg 2012). Aligned reads were discarded. Metagenome assembly was performed with Megahit (v1.2.9) on the remaining valid reads (Li et al. 2015). Open reading frame (ORF) prediction of assembled contigs (≥ 500 bp) was carried out using CD‐HIT (v4.8.1) (Fu et al. 2012) and translated into amino acid sequences. Nonredundant gene sets were constructed for all predicted genes using MMSeqs2 (v13.45111) with clustering parameters of 95% identity and 90% coverage (Steinegger and Söding 2017). Clean reads from each sample were aligned against the nonredundant gene set (95% identity) using Salmon (v1.8.0), and gene abundance in each sample was quantified and normalised to TPM based on the gene length and sequencing depth. The representative sequences (amino acid sequences) were classified and annotated using both the NR database (e‐value ≤ 1e−5) and KEGG database via DIAMOND v2.1.9 (Buchfink et al. 2015). The targeted N and P cycle‐related genes were filtered out based on the main pathways of N and P cycling that simultaneously emerged across all the metagenomic samples. The detail information is shown in Table S12. The abundance of each gene and gene group was calculated by summing the values of all related nonredundant gene catalogues.

### 4.9 Determination of Physiological Characteristics

Leaves at 42 DAG from pot‐grown plants were used to determine the chlorophyll (a/b) content, following the methods described previously (Liu, Xu, et al. 2023). Roots at 42 DAG were washed for subsequent determinations. The nodulation status was recorded and analysed. The total flavonoid content was measured using a Micro Plant Flavonoid Assay Kit. The levels of MDA, O_2_.^−^ and H_2_O_2_ were measured using a content assay kit. The activities of SOD, POD and CAT were measured using enzyme‐linked immunosorbent assay kits. All kits were purchased from Solarbio Science & Technology, Beijing, China. Five biological replicates were performed for each experiment.

### 4.10 Statistics and Data Visualisation

Graphs were generated using R version 4.2.1 and embellished by Adobe Illustrator 2021. The circos plot was generated using the circos (v 0.69‐8). An ANOVA analysis was conducted in R using the ANOVA function in the stats package. Means were compared using Duncan's multiple range test or Student's *t*‐test.

## Author Contributions

S.F., Y.L., G.L. and S.W. designed the experiments. S.F., Y.L. and G.L. prepared the material. S.F. conducted the indoor experiments. J.Z., Z.T. and F.G. conducted the field experiments. S.F., F.X., R.L. and B.B. performed analysis. S.F. wrote the manuscript. Y.L., G.L. and S.W. revised it. All authors read and approved the final manuscript.

## Conflicts of Interest

The authors declare no conflicts of interest.

## Supporting information


**Figure S1.** Dry weight comparison of peanuts under SS and DS conditions.
**Figure S2.** Effects of DNA methylation inhibitor 5‐aza on peanut growth.
**Figure S3.** Impact of 5‐aza on peanut dry weight under SS and DS conditions.
**Figure S4.** Cytosine methylation density of peanut leaf and root under SS and DS conditions.
**Figure S5.** The proportions of methylation levels across different treatments (LD, LS, RD, RS).
**Figure S6.** The distribution of CHH methylation levels across 12 sample groups.
**Figure S7.** Metaplots depict the mean CHH methylation levels of various TE families (*Copia*, DNA transposon, *Gypsy*, LINE, LTR) within each TE and the 2 kb flanking regions.
**Figure S8.** Relationship between DNA methylation and 24 nt‐siRNAs.
**Figure S9.** Relationship between DNA methylation and gene expression.
**Figure S10.** Effect of planting pattern on leaf senescence.
**Figure S11.** Effect of planting pattern on root antioxidant‐related physiological indexes.
**Figure S12.** N, P and K concentration in root.
**Figure S13.** Volcano plots showing DEGs in LD vs. LS (a) and RD vs. RS (b).
**Figure S14.** Phylogenetic analysis of 72 Arabidopsis WRKY proteins and 5 peanut WRKY proteins (red) involved in leaf development by neighbour joining method.
**Figure S15.** Proportion of SNP‐impacted DNA methylation sites across treatments.
**Figure S16.** Correlation analysis of six methylation‐related genes.

**Table S1.** Peanut yield and yield components between SS and DS.
**Table S2.** Raw data quality control statistics and summary of WGBS reads mapping.
**Table S3.** Statistical analysis of CHH methylation levels of various TE families (*Copia*, DNA transposon, *Gypsy*, LINE, LTR) in leaf.
**Table S4.** Statistical analysis of CHH methylation levels of various TE families (*Copia*, DNA transposon, *Gypsy*, LINE, LTR) in root.
**Table S5.** Raw data quality control statistics and summary of RNA‐seq reads mapping.
**Table S6.** Raw data quality control statistics and summary of small RNA‐seq reads mapping.
**Table S7.** Top 10 GO enrichment of overlapping DEGs with DMPs between SS and DS in leaves.
**Table S8.** Details of genes involved in the leaf senescence and flavonoid metabolic processes identified in this study.
**Table S9.** Top 10 GO enrichment of overlapping DEGs with DMPs between SS and DS in roots.
**Table S10.** The rhizosphere soil physicochemical properties.
**Table S11.** Raw data quality control statistics and summary of metagenomic sequencing.
**Table S12.** Information of microbial functional genes involved in the N and P cycling processes identified in this study.
**Table S13.** Summary of SNP‐impacted DNA methylation sites.
**Table S14.** Primers of selected genes for qRT‐PCR analysis.

## Data Availability

The WGBS data are available in the SRA under accession PRJNA1153369, RNA‐seq data under PRJNA1152754 and metagenome data under PRJNA1166702.
