# Supplementary material for: Regulation of DNA Methylation in Peanut Leaves and Roots: Uncovering the Molecular Mechanisms for Increased Yield After Single‐Seed Sowing
Source: Plant Biotechnol J. 2025 Jul 22;23(11):4776–93. doi: 10.1111/pbi.70264 (PMC12576434; doi:10.1111/pbi.70264)
Supplement: Supplementary file 1 — Figure S1. Dry weight comparison of peanuts under SS and DS conditions. Figure S2. Effects of DNA methylation inhibitor 5‐aza on peanut growth. Figure S3. Impact of 5‐aza on peanut dry weight under SS and DS conditions. Figure S4. Cytosine methylation density of peanut leaf and root under SS and DS conditions. Figure S5. The proportions of methylation levels across different treatments (LD, LS, RD, RS). Figure S6. The distribution of CHH methylation levels across 12 sample groups. Figure S7. Metaplots depict the mean CHH methylation levels of various TE families (Copia, DNA transposon, Gypsy, LINE, LTR) within each TE and the 2 kb flanking regions. Figure S8. Relationship between DNA methylation and 24 nt‐siRNAs. Figure S9. Relationship between DNA methylation and gene expression. Figure S10. Effect of planting pattern on leaf senescence. Figure S11. Effect of planting pattern on root antioxidant‐related physiological indexes. Figure S12. N, P and K concentration in root. Figure S13. Volcano plots showing DEGs in LD vs. LS (a) and RD vs. RS (b). Figure S14. Phylogenetic analysis of 72 Arabidopsis WRKY proteins and 5 peanut WRKY proteins (red) involved in leaf development by neighbour joining method. Figure S15. Proportion of SNP‐impacted DNA methylation sites across treatments. Figure S16. Correlation analysis of six methylation‐related genes. [file PBI-23-4776-s002.docx]

**Figure S1.** Dry weight comparison of peanuts under SS and DS Conditions

(a) stem dry weight, (b) leaf dry weight, (c) root dry weight. Treatments are same as Fig.1. Data are presented as mean ± SD (a-b, *n* = 5 pots per treatment; c, *n* = 6-8 pots per treatment). Significant differences marked by asterisks between SS and DS in same time (*p* < 0.05, Student’s *t* test).

**Figure S2.** Effects of DNA methylation inhibitor 5-aza on peanut growth.

Peanut seedlings were grown under SS conditions spaying 0, 10, 20 and 30 µM 5-aza from 28 to 42 DAG. Data are Means ± SD (n = 6). Different letters indicate a statistical difference at *p* < 0.05.

**Figure S3.** Impact of 5-aza on peanut dry weight under SS and DS conditions.

(a) stem dry weight per plant, (b) leaf dry weight per plant, (c) root dry weight per plant. Treatments are same as Fig.2. Data are presented as mean ± SD (a-b, *n* = 5 pots per treatment; c, *n* = 6-8 pots per treatment). Different letters above bars indicate significant differences (p < 0.05).


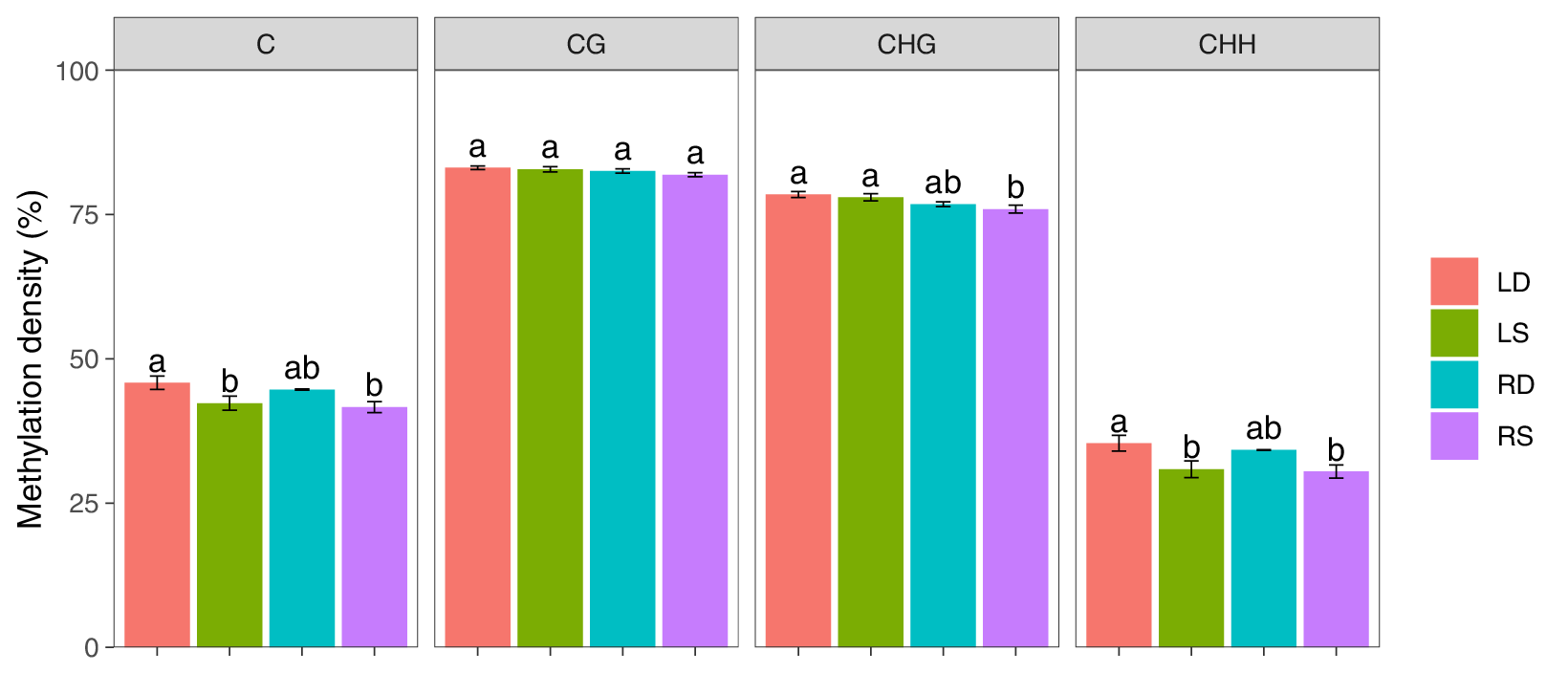


**Figure S4.** Cytosine methylation density of peanut leaf and root under SS and DS conditions.

LD, peanut leaf in DS; LS, peanut leaf in SS; RD, peanut root in DS; RS, peanut root in SS. Data are Means ± SD (n = 3). Different letters above bars indicate statistically significant differences (p < 0.05).


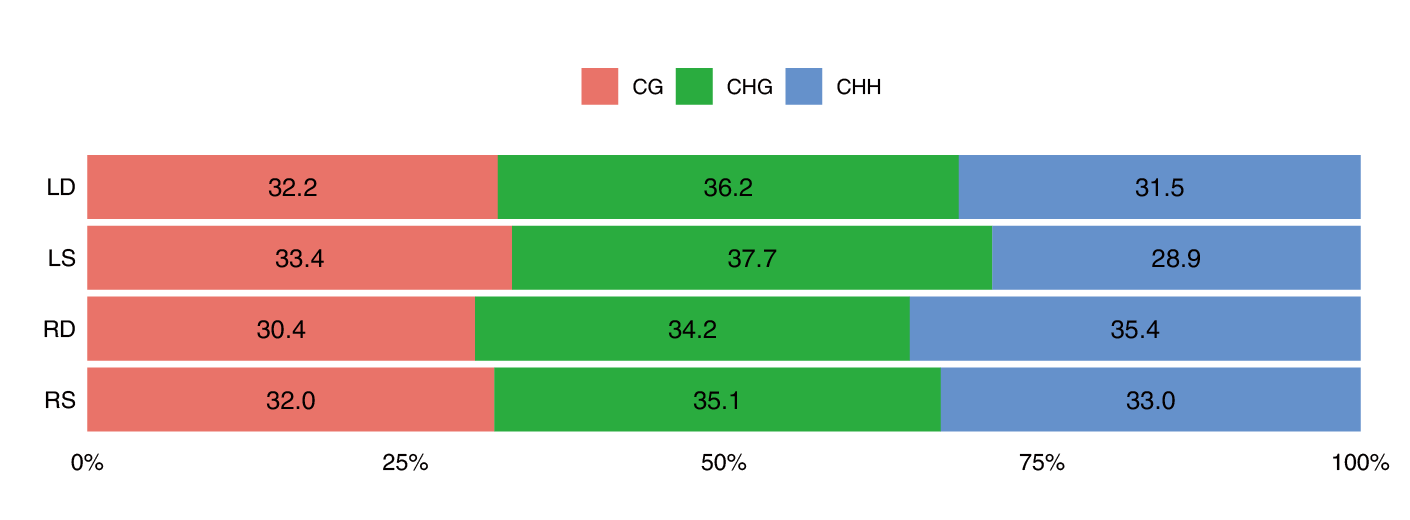


**Figure S5.** The proportions of methylation levels across different treatments (LD, LS, RD, RS).

Colors represent different contexts (CG, CHG, CHH). The mean value of three replicates was adopted.


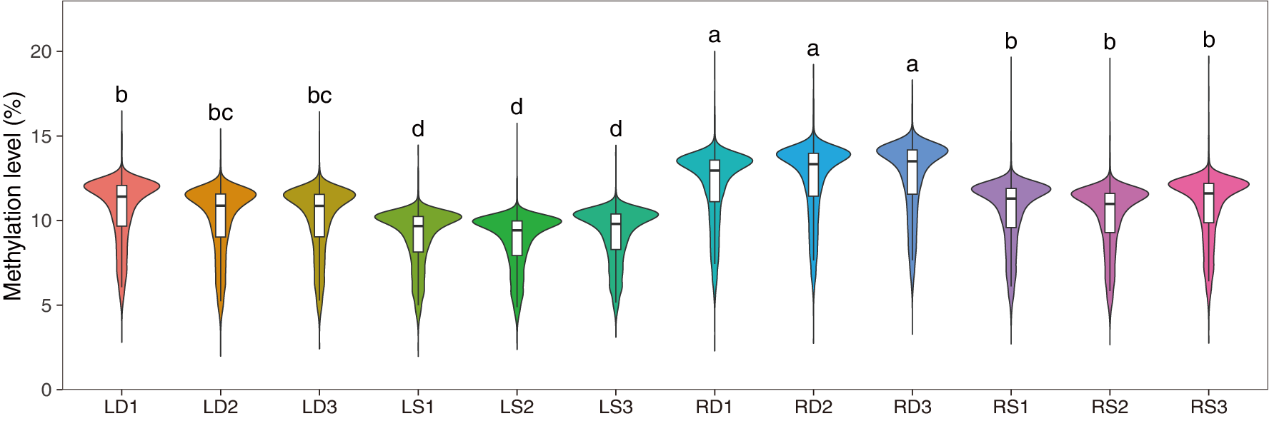


**Figure S6.** The distribution of CHH methylation levels across 12 sample groups.

The values were measured using a 1-Mb window, same to Figure 3d. The identical and different letters represent nonsignificant and significant differences (*P* < 0.05).


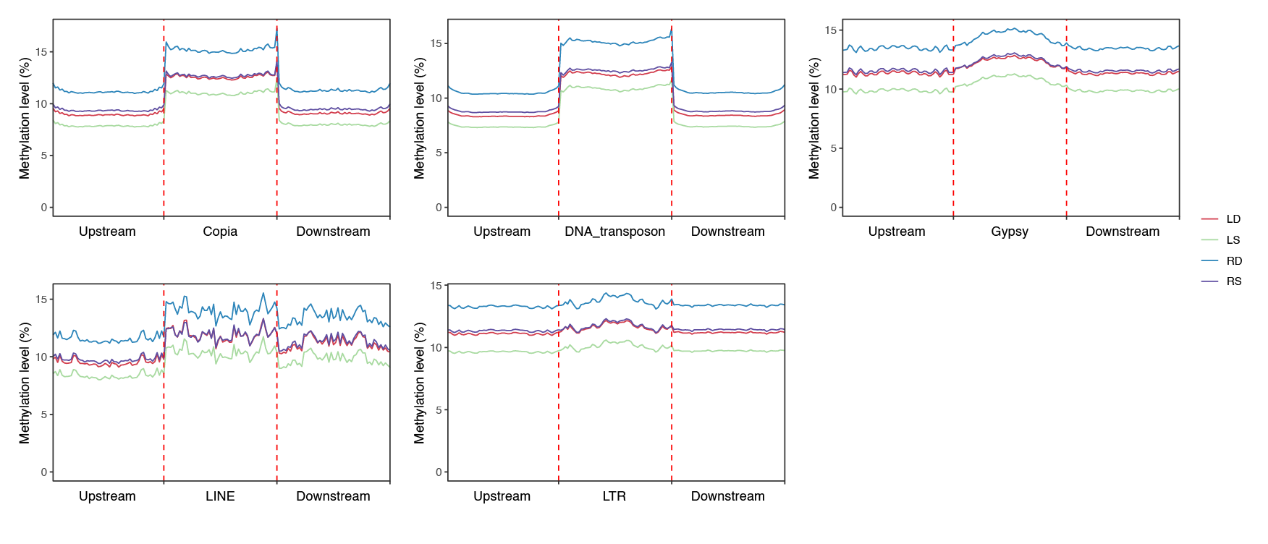
**Figure S7.** Metaplots depict the mean CHH methylation levels of various TE families (*Copia*, DNA transposon, *Gypsy*, LINE, LTR) within each TE and the 2 kb flanking regions.

Treatments are the same as in Figure 3c.


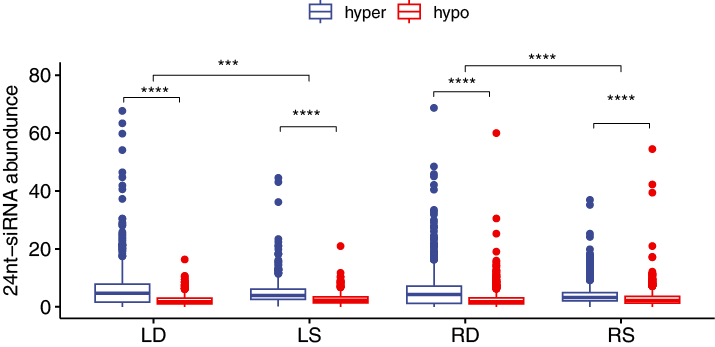


**Figure S8.** Relationship between DNA methylation and 24nt-siRNAs.

Abundance of 24-nt siRNAs located in the CHH hypermethylated and hypomethylated regions (****P*-value < 0.001, *****P*-value < 0.0001, Student's *t* test).


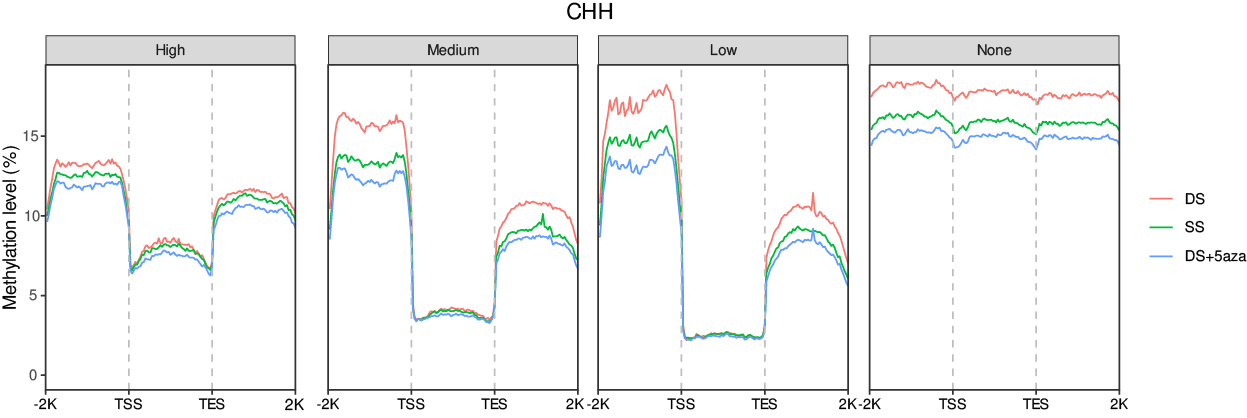


**Figure S9.** Relationship between DNA methylation and gene expression.

CHH methylation levels of genes grouped by expression levels (none, low, moderate, high) in DS, SS, and DS+5-aza treatments.


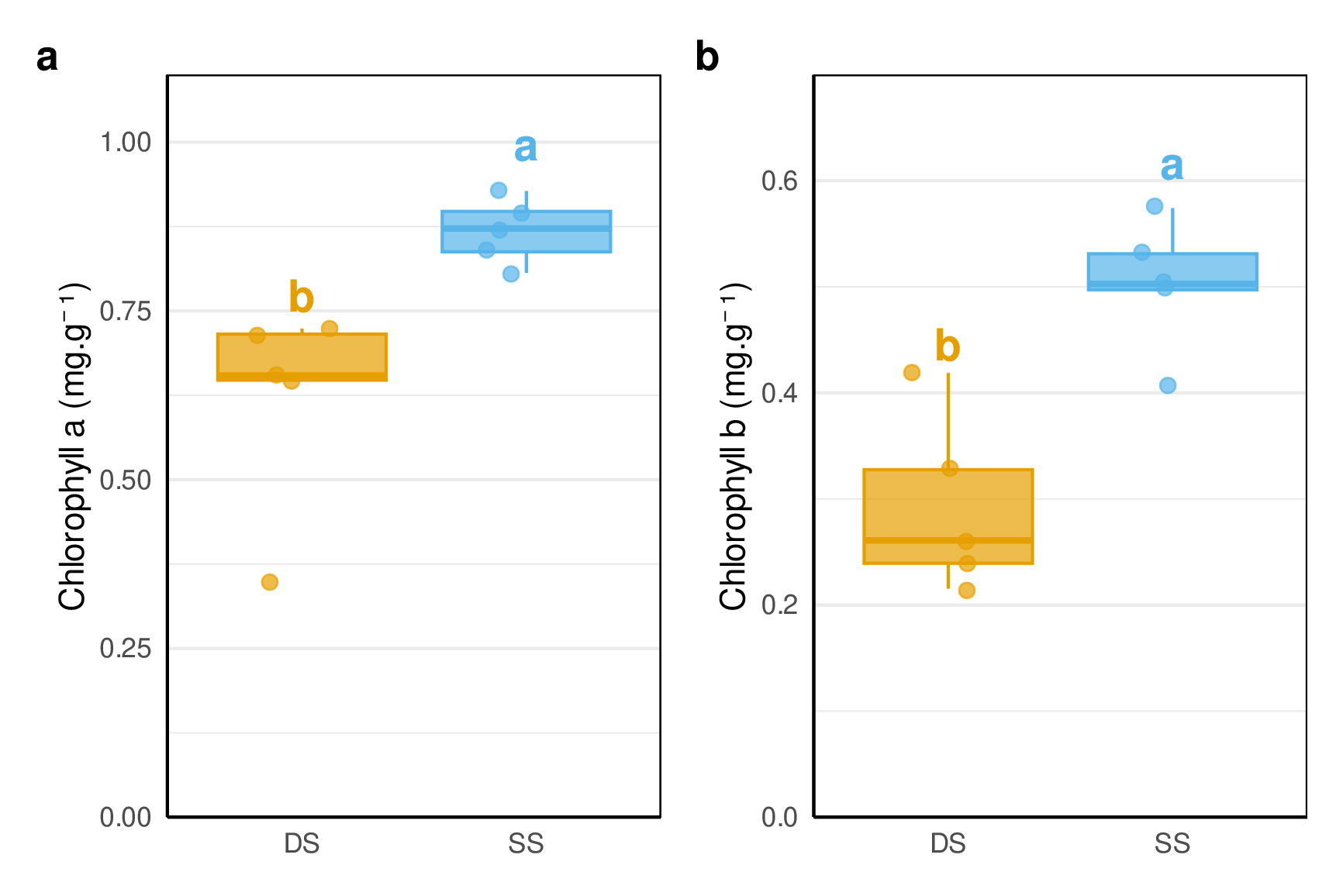


**Figure S10.** Effect of planting pattern on leaf senescence.

(a) Chlorophyll a, (b) Chlorophyll b. n=5. Different letters indicate significant differences among treatments (*P* < 0.05, Student’s *t*-test).


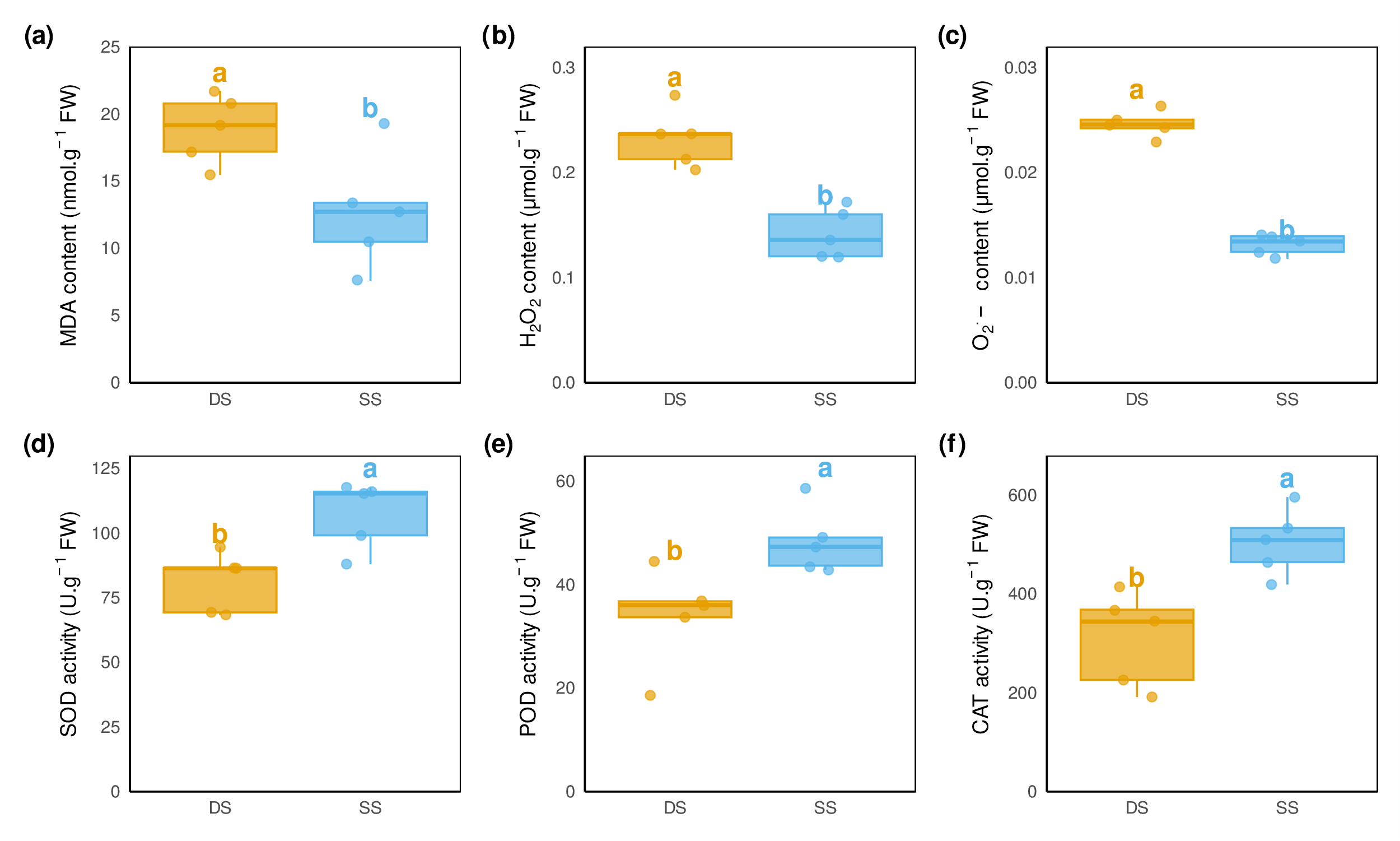


**Figure S11.** Effect of planting pattern on root antioxidant-related physiological indexes.

(a) MDA content, (b) H_2_O_2_ content, (c) O_2_^.-^ content, (d) SOD activities, (e) POD activities, (f) CAT activities. n=5. Different letters indicate significant differences between treatments (*P* < 0.05, Student’s *t*-test).

**Figure S12.** N, P and K concentration in root.

Asterisk indicate significant differences among treatments (**P* < 0.05, Student’s *t*-test).


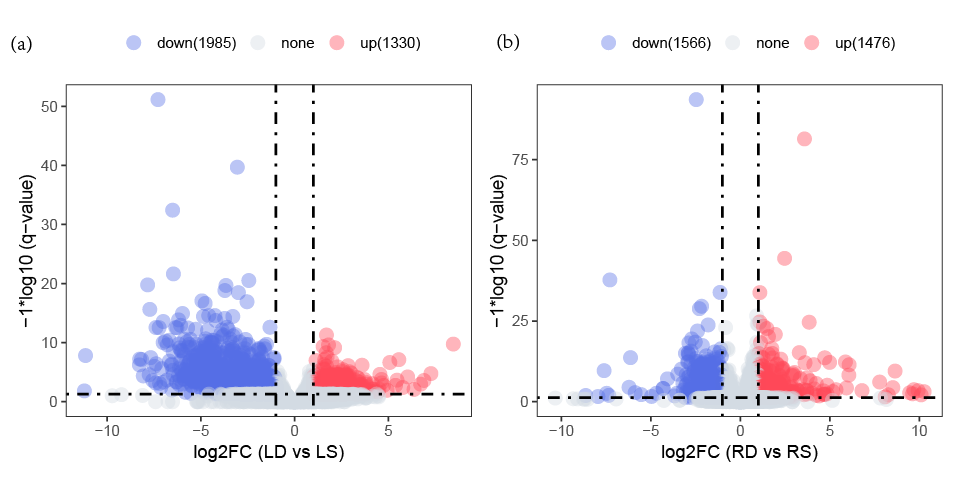


**Figure S13.** Volcano plots showing DEGs in LD vs LS (a) and RD vs RS (b).

(a) Comparison between peanut leaf in double-seed (LD) and peanut leaf in single-seed (LS) conditions. (b) Comparison between peanut root in double-seed (RD) and peanut root in single-seed (RS) conditions. Blue points indicate significantly downregulated genes, red points indicate significantly upregulated genes, and gray points represent genes with no significant change. Numbers in parentheses indicate the total count of significantly regulated genes in each direction.


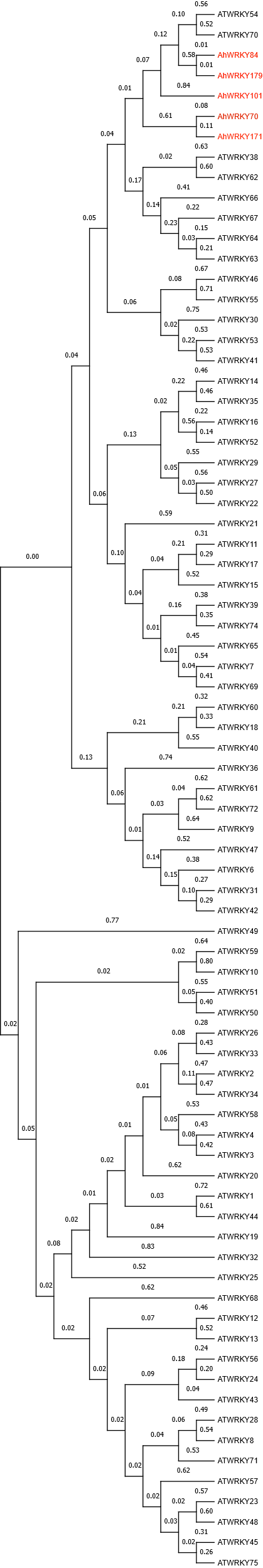


**Figure S14.** Phylogenetic analysis of 72 Arabidopsis WRKY proteins and 5 peanut WRKY proteins (red) involved in leaf development by neighbor joining method.


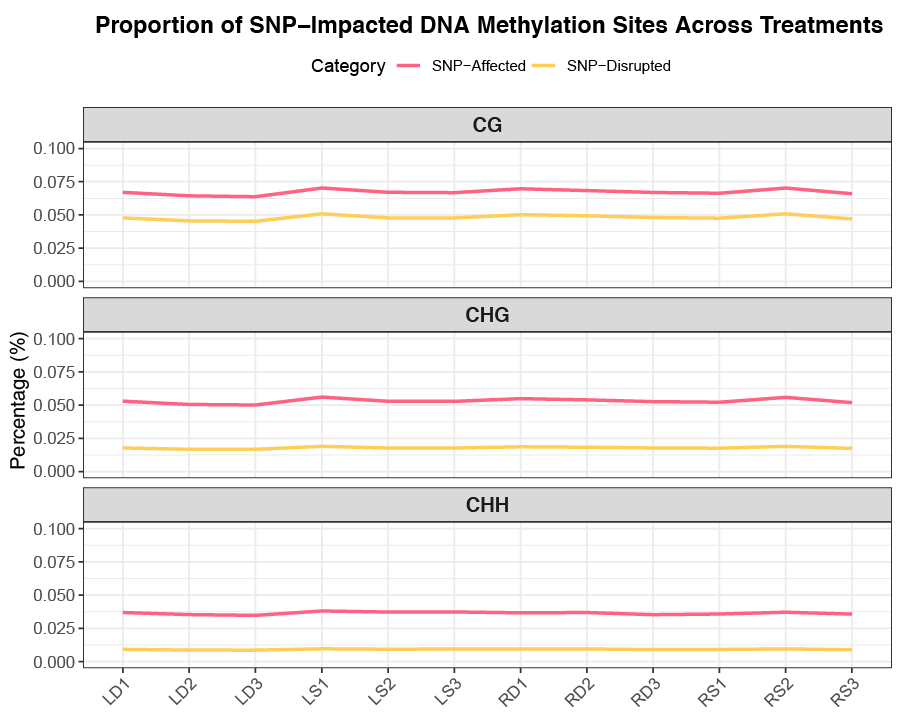


**Figure S15.** Proportion of SNP-impacted DNA methylation sites across treatments.

Line plots showing the percentage of methylation sites affected by single nucleotide polymorphisms (SNPs) across various treatment conditions. Data are presented for three cytosine methylation contexts: CG (top panel), CHG (middle panel), and CHH (bottom panel), where H represents A, T, or C. Pink lines indicate SNP-affected sites, while yellow lines represent SNP-disrupted sites. LD, peanut leaf in double-seed; LS, peanut leaf in single-seed; RD, peanut root in double-seed; RS, peanut root in single-seed.


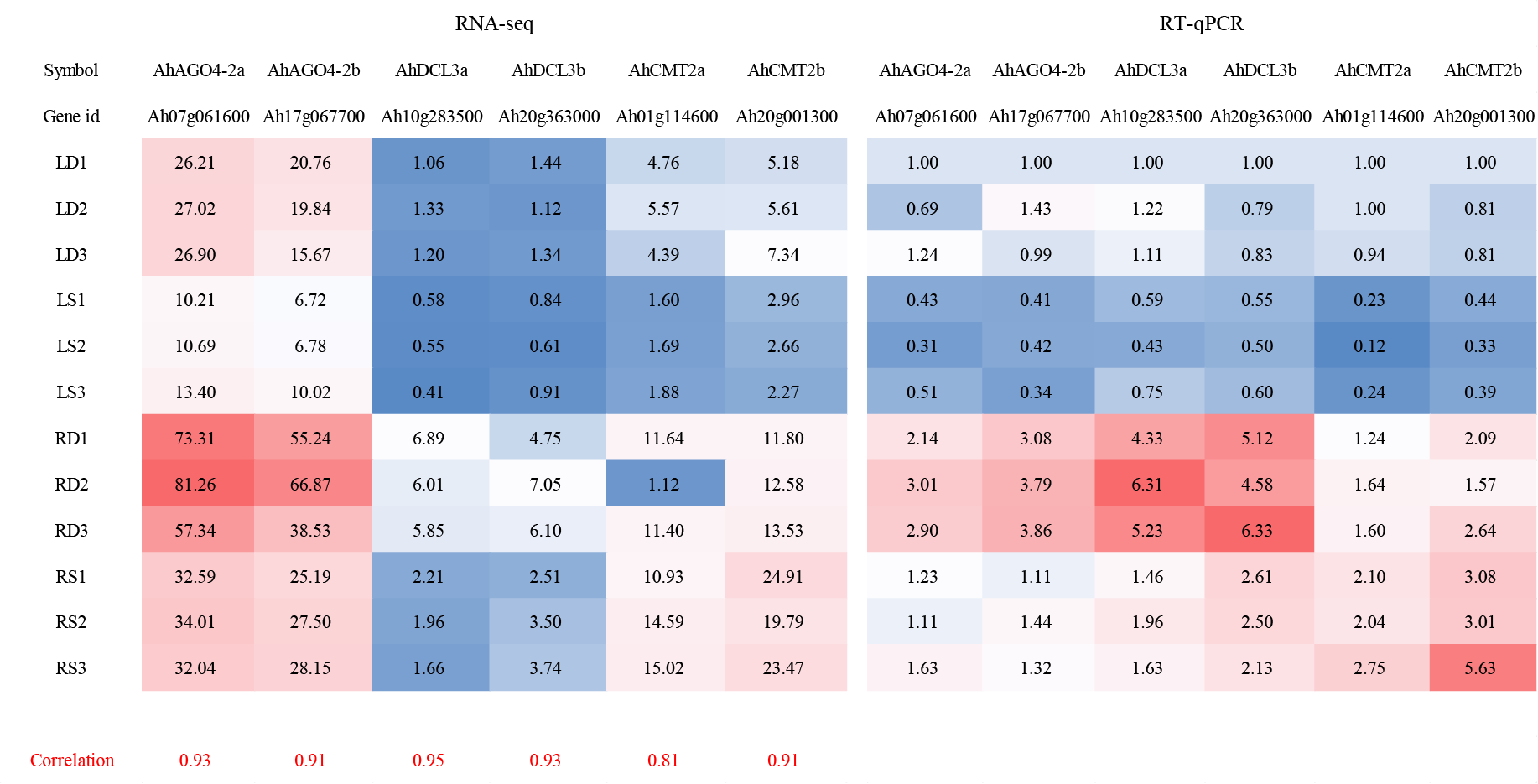


**Figure S16.** Correlation analysis of six methylation-related genes.

The values beneath the two heatmaps represent the Pearson correlation coefficients, which were calculated in Excel. RNA-seq data are presented as TPM values. For RT-qPCR, the value of LD1 was set to "1". The heatmaps use a red-to-blue gradient to indicate values from high to low.
